# Efficacy of Vafidemstat in Experimental Autoimmune Encephalomyelitis Highlights the KDM1A/RCOR1/HDAC Epigenetic Axis in Multiple Sclerosis

**DOI:** 10.3390/pharmaceutics14071420

**Published:** 2022-07-06

**Authors:** Fernando Cavalcanti, Elena Gonzalez-Rey, Mario Delgado, Clara P. Falo, Leyre Mestre, Carmen Guaza, Francisco O’Valle, Michele M. P. Lufino, Jordi Xaus, Cristina Mascaró, Serena Lunardi, Natalia Sacilotto, Paola Dessanti, David Rotllant, Xavier Navarro, Mireia Herrando-Grabulosa, Carlos Buesa, Tamara Maes

**Affiliations:** 1 Oryzon Genomics S.A., Carrer Sant Ferran 74, 08940 Cornellà de Llobregat, Spain; fcavalcanti@oryzon.com (F.C.); mlufino@oryzon.com (M.M.P.L.); jxaus@oryzon.com (J.X.); cmascaro@oryzon.com (C.M.); nsacilotto@oryzon.com (N.S.); pdessanti@oryzon.com (P.D.); drotllant@oryzon.com (D.R.); cbuesa@oryzon.com (C.B.); 2 Institute of Parasitology and Biomedicine López-Neyra, IPBLN-CSIC, PTS-Granada, 18016 Granada, Spain; elenag@ipb.csic.es (E.G.-R.); mdelgado@ipb.csic.es (M.D.); 3 Department of Functional and Systems Neurobiology, Cajal Institute (CSIC), 28034 Madrid, Spain; mestre.leyre@gmail.com (L.M.); cgjb@cajal.csic.es (C.G.); 4 Department of Pathology, School of Medicine, IBIMER and IBS-Granada, Granada University, 18071 Granada, Spain; fovalle2013@gmail.com; 5 Departament de Biologia Cellular, Fisiologia i Immunologia, Institut de Neurociències, Universitat Autònoma de Barcelona, and Centro de Investigación Biomédica en Red sobre Enfermedades Neurodegenerativas (CIBERNED), 08193 Barcelona, Spain; xavier.navarro@uab.cat (X.N.); mireia.herrando@uab.cat (M.H.-G.)

**Keywords:** epigenetics, KDM1A, vafidemstat, multiple sclerosis, neuroinflammation

## Abstract

Lysine specific demethylase 1 (LSD1; also known as KDM1A), is an epigenetic modulator that modifies the histone methylation status. KDM1A forms a part of protein complexes that regulate the expression of genes involved in the onset and progression of diseases such as cancer, central nervous system (CNS) disorders, viral infections, and others. Vafidemstat (ORY-2001) is a clinical stage inhibitor of KDM1A in development for the treatment of neurodegenerative and psychiatric diseases. However, the role of ORY-2001 targeting KDM1A in neuroinflammation remains to be explored. Here, we investigated the effect of ORY-2001 on immune-mediated and virus-induced encephalomyelitis, two experimental models of multiple sclerosis and neuronal damage. Oral administration of ORY-2001 ameliorated clinical signs, reduced lymphocyte egress and infiltration of immune cells into the spinal cord, and prevented demyelination. Interestingly, ORY-2001 was more effective and/or faster acting than a sphingosine 1-phosphate receptor antagonist in the effector phase of the disease and reduced the inflammatory gene expression signature characteristic ofEAE in the CNS of mice more potently. In addition, ORY-2001 induced gene expression changes concordant with a potential neuroprotective function in the brain and spinal cord and reduced neuronal glutamate excitotoxicity-derived damage in explants. These results pointed to ORY-2001 as a promising CNS epigenetic drug able to target neuroinflammatory and neurodegenerative diseases and provided preclinical support for the subsequent design of early-stage clinical trials.

## 1. Introduction

KDM1A (LSD1) is a flavin adenine dinucleotide dependent amine oxidase that is located in the nucleus and acts as a histone modifying enzyme [1]. KDM1A is recruited into repressive transcription complexes, interacts tightly with RCOR and HDAC1/2 [2], and demethylates primarily, although not exclusively, H3K4me2/1 marks [3,4,5], associated with the active transcription state [6]. However, KDM1A also acts as a scaffold protein for the assembly of multi-protein complexes needed to control transcription and exerts part of its cellular functions independent of its catalytic activity [7].

KDM1A expression starts at early embryonic stages and persists during adult life. The protein is expressed in many tissues and required in multiple biological processes including early embryo development [8], hematopoiesis [9], neurogenesis, and terminal neuronal differentiation [10,11,12,13]. However, the protein is also involved in human diseases. Consequently, KDM1A inhibitors have emerged as a promising therapy in oncology [14,15,16], and more recently, they have been proposed for treatment of neurodevelopmental and CNS diseases. For example, iadademstat (ORY-1001) and tranylcypromine (TCP) reversed schizophrenia-related phenotypes in SetD1a deficient mice [17], TAK-418 restored adult neurogenesis, and improves hippocampal memory in a mouse model of Kabuki syndrome [18], and TCP and GSK2879552 blocked cell death and induced neuroprotective genes in a retinitis pigmentosa mouse model [19].

Vafidemstat (ORY-2001), an orally bioavailable brain penetrant inhibitor of KDM1A, is in clinical development for treatment of CNS diseases. Originally identified in vitro as a dual inhibitor of KDM1A and monoamine oxidase B (MAO-B), the compound inhibits only KDM1A at therapeutic doses in vivo [20,21]. ORY-2001 was shown to improve learning and reduce the memory deficit in the SAMP8 (Senescence Accelerated Mouse Prone 8) model for accelerated aging and Alzheimer’s Disease (AD) to reduce pathologically aggressive behavior and to improve other social behavior alterations in rodent models. Interestingly, ORY-2001 also reduced the inflammation signature in the hippocampus of SAMP8 mice, including S100A9 and T-cell receptor b genes [22].

Upregulation of S100A9 has been described both in Multiple sclerosis (MS) [23] and in Experimental Autoimmune Encephalomyelitis (EAE) mice [24]. Furthermore, compounds that disrupt the binding of S100A9 to the Toll-Like Receptor 4 (TLR4) were shown to inhibit acute EAE in mice [25], illustrating the relevance of this biomarker to MS.

MS is the most prevalent chronic neuroimmune disease and is currently incurable [26]. It is a disabling neuro-degenerative disease characterized by an autoimmune response against myelin sheaths that surround central axons, thus resulting in demyelination and failure of impulse conduction, progressive axonal loss, and profound dysfunctions of the CNS [27]. Neurodegeneration and neuroinflammation are key components in the progression and chronicity of this disease. Despite intensive investigation, the mechanisms of disease pathogenesis remain unclear, and curative therapies are unavailable for MS. EAE shows pathologic and clinical similarities to human MS and is used as a model system to test potential therapeutic agents. Both EAE and MS are considered archetypical CD4 Th1 cell-mediated autoimmune diseases in which Th1 cells reactive to components of the myelin sheath, infiltrate the CNS parenchyma, release proinflammatory cytokines and chemokines and promote macrophage infiltration and activation. Inflammatory mediators such as cytokines (i.e., IL-12, IN-γ and TNF-α), nitric oxide, and free radicals, produced by infiltrating cells and resident microglia, play a critical role in demyelination, contributing to oligodendrocyte loss and degenerative axonal pathology [28]. In addition to physical disability, it has become clear that cognition and behavioral problems such as aggression and others are also common in MS patients [29]. Although the etiology of the disease is unknown, an epigenetic component appears to influence the onset and progression of the disease, and in addition to DNA methylation and miRNA-based gene expression regulation, histone modification has also been implicated in MS pathogenesis [30].

Current data support the relevance of KDM1A as a potential drug target for brain and neurological disorders, but its precise role in neuroinflammation has not been elucidated. The aim of this study was to evaluate the efficacy and mechanism of action of the KDM1A inhibitor ORY-2001 in mouse models mimicking the chronic stage of MS. 

## 2. Materials and Methods

### 2.1. Reagents and Resources

Details of all reagents and resources used in this research are included in Supplementary Materials Table S4. In vitro potencies of inhibitors used were the following: ORY-2001 (IC_50_ KDM1A = 101 ± 40 nM; IC_50_ MAO-B = 73 ± 34 nM), ORY-LSD1 (IC_50_ KDM1A = 10 ± 3 nM; IC_50_ MAO-B > 100 μM). The dose used rasagiline (Waterstone, Wuhan, China) (IC_50_ KDM1A > 100 μM; IC_50_ MAO-B = 69 ± 0.5 nM) or FTY720 (Cayman Chemical, Ann Arbor, MI, USA).

### 2.2. Animals and Ethic Statement

All of the studies reported in this research followed the institutional guidelines for the care and protection of animals used for scientific purposes (European Communities Council Directive 2010/63/EU), were approved in accordance with national (Royal Decree 53/2013 BOE No. 34) guidelines and by the corresponding ethical committees, and were reported to be in compliance with the ARRIVE v.2.0 guidelines [31]. Specifically, the procedures using the EAE model were approved sequentially by the Ethical Committee for animal experimentation at the IPBLN (protocol number CEEA-IPBLN-2016/12) by the Ethical Committee for Animal Experimentation at the CSIC (superior authorized body, protocol number 637/2017) and validated by the Ethical Committee for Animal Experimentation of the Junta de Andalucia with protocol number 13/04/2018/049. Studies using the Theiler’s murine encephalomyelitis virus TMEV-induced model were approved by the Ethics Committee on animal experimentation at the CSIC under protocol number 2013/03 CEEA-IC. The experiments reported for spinal cord organotypic cultures and chronic excitotoxicity treatment using Sprague–Dawley pups were approved by the Ethics Committee of Universitat Autònoma de Barcelona under procedure CEEAH1963M. Female C57BL/6 (EAE experiments) and SJL/J mice (TMEV studies) were supplied by Charles River and maintained in the Animal Units of IPBLN (Granada, Spain) and Cajal Institute (Madrid, Spain), respectively. Sprague–Dawley rats and pups were kept and obtained from the UAB Servei dÉstabulari breeding colony (Barcelona, Spain). All animals were housed in cages in specific pathogen free conditions with a controlled temperature/humidity environment (21 ± 2 °C, 50–70% relative humidity). The light/dark cycle was maintained at 12:12 h (lighting on at 7:00 a.m.). Adult animals were fed with rodent chow (Global Diet 2018, Harlan, Barcelona, Spain) and tap water ad libitum. Mice were randomly assigned to the different experimental groups (10 and 6 mice per group and cage, respectively, for EAEand TMEV experiments; 5 rat pups per group and cage, to obtain material for spinal cord organotypic culture).

### 2.3. Induction and Treatment of Experimental Autoimmune Encephalomyelitis (EAE) Model

**Figure 1:**
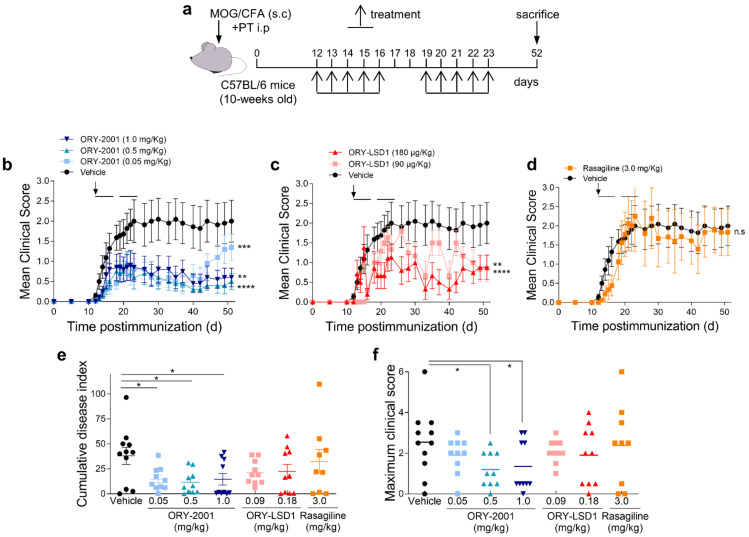
Treatment with ORY-2001 reduces clinical symptoms and EAE severity. (**a**) Chronic progressive EAE was induced in C57BL/6 mice by immunization with MOG_35–55_. Immunized animals were treated orally during two periods of five days (horizontal bars in (**b**–**d**)) with vehicle, with ORY-2001 ((**b**), at 1.0, 0.5, or 0.05 mg/kg), with ORY-LSD1 ((**c**), at 180 or 90 µg/kg) or with rasagiline (d, 3.0 mg/kg) starting at the onset on day 12 (pointed by an arrow in (**b**–**d**)) and sacrificed 52 days after immunization; (**b**–**d**) the data represent the progression of the disease evaluated as the mean clinical score. ** *p* < 0.01, *** *p* < 0.0005, **** *p* < 0.0001 between vehicle and treated mice. n.s: no statistical significance; (**e**) the cumulative disease index (the sum of the clinical scores reached for each animal every day until day 51 post-immunization); (**f**) the maximum clinical score reached by each animal in any day during the entire period investigated are shown in each experimental group (mean and individual scores). N = 11 mice/group for vehicle; N = 10 mice/group for ORY-2001 treatment; N = 10 mice/group for ORY-LSD1 treatment, and N = 9 mice/group for rasagiline treatment. * *p* < 0.05 between vehicle and ORY-2001-treated mice. All data represent mean ± SEM.

To induce chronic EAE, C57BL/6 mice (8 weeks old, 25–30 gr) were immunized s.c. with 100 µg of the myelin oligodendrocyte glycoprotein peptide MOG_35–55_ (GeneScript) emulsified in complete Freund adjuvant containing 4 mg/mL Mycobacterium tuberculosis H37 RA (Difco) (Figure 1a). Mice also received i.p. injections of 200 ng of pertussis toxin on days 0 and 2 [32]. Immunized mice were randomly distributed in different groups. Treatment consisted of the administration of ORY-2001 (1, 0.5 and 0.05 mg/kg), ORY-LSD1 (0.18, 0.09 and 0.06 mg/kg), FTY720 (1 mg/kg), or rasagiline (3 mg/kg) by oral gavage starting after the onset of the disease (day 12 post-immunization). The dose range used for ORY-2001 and ORY-LSD1 was a function of their relative in vivo potency of KDM1A inhibition of each compound. The dose chosen for in vivo treatment of mice with rasagiline is sufficient for full inhibition of MAO-B and confers full protection toward a 1-methyl-4-phenyl-1,2,3,6-tetrahydropyridine (MPTP) insult in mice. MPTP is a prodrug which is converted to the neurotoxin 1-methyl-4-phenylpyridinium (MPP+) by MAO-B in vivo. The dose chosen for fingolimod (FTY720) was the highest dose used in previously reported efficacy studies and provided full efficacy in an EAE model in mice [33,34]. 

Compounds were administered once daily for five consecutive days in two cycles, from day 12 to 16 and from day 19 to 23 post-immunization. Control mice were treated with vehicle (2% *v*/*v* Tween-80 + 98% HPβCD (13% *w*/*v*)) following the same administration regime. HPβCD was included to facilitate dissolution. Mice were scored daily for signs of EAE according to the following clinical scoring system: 0, no clinical signs; 0.5, partial loss of tail tonicity; 1, complete loss of tail tonicity; 2, flaccid tail and abnormal gait; 3, complete hind leg paralysis; 4, hind leg paralysis with hind body paresis; 5, hind and foreleg paralysis; and 6, death. Intermediate phenotypes were scored as a half integer. Animals were sacrificed when they kept a score of 5 more than 24 h. Investigators which administered the compounds and scored the animals were blinded to the treatments.

### 2.4. Induction and Treatment of Theiler’s Murine Encephalomyelitis Virus (TMEV) Model

Susceptible SJL/J mice (6 weeks old), previously anesthetized with isoflurane, were inoculated in the cerebral parenchyma with 2 × 10^6^ plaque forming units (pfu) of the Daniel’s strain of TMEV, diluted in 30 μL of Dulbecco’s Modified Eagle Medium (DMEM) supplemented with 5% fetal bovine serum (FBS). The injection takes place in the right hemisphere of the cerebral cortex using a Hamilton syringe coupled to a pipette tip in such a way that it allows for an exposure of the needle of about 2–3 mm; thus, the virus is always injected at the same depth. Sham animals are subjected to the same protocol, but they only received 30 µL of DMEM supplemented with 5% fetal bovine serum. Treatment consisted of the administration of ORY-2001 by oral gavage at the dose of 0.3 and 1.0 mg/kg, starting at the onset of the clinical signs (day 72 post-infection), once a day for five consecutive days from day 72 to day 76 and from day 79 to day 83 post-infection. Control mice were treated with vehicle [2% *v*/*v* Tween-80 + 98% HPβCD (13% *w*/*v*)] following the same administration regime (Figure 4a). The animals were weighed every week from the onset of infection and clinical signs were evaluated using the criterion described by Moses Rodriguez (Mayo Clinic), source of the Daniel’s strain of TMEV to assess the severity of the disease by the general appearance: Score 1 = Shaggy and scruffy hair; 2 = scruffy appearance and hunched back; 3 = no spontaneous movement, reduced induced activity; and Gait: Score 1 = mild ataxia with inconsistent waddling gait; 2 = moderate ataxia with consistent waddling gait; 3 = severe ataxia with reduced righting response; 4 = spastic paresis of hind legs. The score of the animals and motor behavior were analyzed by trained observers blind to treatment and experimental groups.

### 2.5. Evaluation of Motor Function in TMEV Model

The screening for locomotor activity was performed by analyzing spontaneous motor activity using an Activity Cage (Activity Monitor System Omnitech Electronics, Inc., Columbus, OH, USA) and by evaluating the mice performance in the Rotarod test. The activity cage consists of a four-compartment metacrylate cage, surrounded by sensors in such a way that it registers the movements of the mouse in both vertical and horizontal position and ambulation. The test consists of introducing the mice into two compartments of the cage placed diagonally so as not to interfere with the sensors of the other mouse. During two five-minute cycles (0–5 min, 5–10 min), horizontal activity (HACTV) or vertical activity (VACTV) are recorded. The first cycle evaluates the spontaneous activity in a new environment and the second measures the activity of the animal once habituated to the new environment. This task was performed on days 60, 70, 78, and 86 post-infection. The rotarod (Ugo Basile, Milan, Italia) consists of a rotating cylinder (roll) suspended above a cage floor turning at a constant speed or acceleration. The system is completed with a stopwatch for each compartment that stops when the mouse falls from the roll to a lever on the bottom of the device. One week prior to the test, mice were trained and familiarized with the test for one minute at a constant speed. The length of time that mice stay on the rotating roll is a measure of their balance and motor coordination. The test consists of measuring the time that mice remain on the roll with constant acceleration for a maximum time of five minutes. Mice that developed strategies to stay on the roll (p.e. seeking support on the wall) were excluded from evaluation. The rotarod test was performed on day 72, 78, and 86 post-infection.

### 2.6. Tissue Collection and Cell Isolation

For mechanistic studies in the EAE model, animals were divided into two groups, and samples were collected on day 26 post-immunization (sub-chronic phase), 3 days after the last dose, or on day 17 post-immunization for evaluating the effector phase. For this, mice were euthanized by intracardiac perfusion with saline (0.9%NaCl in H_2_O MilliQ) after pentobarbital anesthesia (Doletal), and blood samples, spleen, draining lymph nodes (DLNs: cervical, inguinal and axillary), brain, and spinal cord were removed. Serum samples were used for autoantibodies determination. Single-cell suspensions were obtained from spleen or pooled DLNs and used for the determination of cell number and auto-reactive and inflammatory responses, as described later. Brain and spinal segments of the cervical and lumbar regions were prepared separately and processed for RNA isolation, protein extraction, and histopathological analysis. Proteins were extracted from cervical and lumbar segments of spinal cord and brain from EAE mice via homogenization (50 mg tissue/mL) in lysis buffer (50 mM Tris-HCl, pH 7.4, 0.5 mM DTT, and 10 µg/mL proteinase inhibitors PMSF, pepstatin, and leupeptin). Samples were centrifuged (20.000× *g*, 15 min, 4 °C), and the supernatants were assayed for protein concentration (using Bradford method) and for cytokine/chemokine contents by using specific sandwich ELISAs according to manufacturer’s recommendations (BD Bioscience, Heidelberg, Germany and Peprotech, London, UK). In the TMEV model on day 86 after infection, mice were anesthetized and perfused transcardially, as described above. Spinal cords were obtained via extrusion with saline and prepared for further study. 

### 2.7. Histopathological Analysis

Cervical and lumbar spinal cord segments of spinal cords obtained from EAE mice sacrificed on day 17 or 26 post-immunization were divided and processed for inclusion and sectioning in paraffin and for inclusion in an optimal cutting temperature (OCT) compound and future cryosectioning of the samples. Spinal cord segments were immediately fixed with buffered 10% formalin for 48 h, dehydrated and included in paraffin using standard techniques. For a preliminary analysis, transversal sections (4 µm thickness) were stained with Luxol fast blue, cresyl violet, and hematoxylin following the Klüver–Barrera technique and were analyzed for the presence of areas of demyelination and cell infiltration using a light microscope (Leica, DM2000). After hematoxylin/eosin staining in the TMEV model, cell infiltration in the cervical and thoracic spinal cord was evaluated according to the criteria of Mansilla et al. [35]: score 0: no lesion; 1: cellular infiltration only in the meninges; 2: very discrete and superficial infiltrates in the parenchyma; 3: moderate infiltration (less than 25%) in the white matter; 4: severe infiltration (less than 50%) in the white matter; and 5: high severe infiltration (>50%) in the white matter. The remaining spinal cord segments from EAE mice and from mice of the TMEV model were fixed with 4% paraformaldehyde/0.1 M phosphate buffer pH 7.4 for 12 h at 4 °C, cryopreserved in 30% sucrose in PBS for 24 h, embedded in OCT in liquid nitrogen, and stored at −80 °C prior to cryosectioning and immunohistochemistry. 20 and 30 µm-thick transversal cryostat sections from Tissue Tek embedded tissue were obtained from EAE and TMEV samples, respectively, and kept in a cryoprotection solution (30% ethylene glycol, 10 mg/mL polyvinylpyrrolidone-40 (PVP-40), 300 mg/mL sucrose in 60 mM PB) at −20 °C. Mouse #6 from the TMEV + vehicle group died on day 77 post-infection and was therefore not included. 

### 2.8. Flow Cytometry Analysis

For the identification of naïve, memory, and effector T cells, lymph nodes were isolated from different EAE groups at the peak of the disease. Cells were collected and distributed into polystyrene tubes for flow cytometry at 0.5 *×* 10^6^ cells/tube and washed twice with washing buffer (10% FBS, 1% sodium azide in PBS; 300 g 5 min 4 °C). Before specific staining, cells were blocked with mouse BD Fc block to avoid nonspecific binding to Fc- receptors and marked with 7-AAD (7-amino-actinomycin D, Calbiochem, San Diego, CA, USA) to distinguish dead cells. Then, cells were incubated with PerCP/Cy5.5 conjugated anti-CD4, APC conjugated anti- CD62L (2 µg/mL), and PE conjugated anti-CD44 mAbs (0.5 µg/mL), following manufacturer’s recommendations (BioLegend) (Supplementary Materials Table S4). Briefly, after washing, cells were stained for 30 min at 4 °C, washed again, and analyzed in a FACSCalibur flow cytometer (BD Pharmingen, Le Pont-de-Claix, France). We used isotype-matched antibodies as controls.

### 2.9. Determination of Autoreactive Response

Spleen and draining lymph node (DLN) cells (10^6^/mL) collected from EAE mice were stimulated in complete medium (RPMI 1640 containing 10% FBS, 50 µM 2-ME, 2 mM L-glutamine, 100 U/mL penicillin, and 100 µg/mL streptomycin) with 15 µM MOG_35–55_. After 48 h, cytokine (IL-4, TNF-alpha, IFN-gamma) and chemokine (IP-10 and MCP1) contents in culture supernatants were determined by sandwich ELISAs using capture/biotinylated detection Abs according to the manufacturer’s recommendations. The antibodies used are indicated in Supplementary Materials Table S4. Cell proliferation was evaluated after 72 h by adding 2.5 µCi/mL tritiated-thymidine during the last 8 h of culture and determining cpm incorporation in a Microbeta counter. We used T cell activation with an anti-CD3 molecular antibody (1 µg/mL) or ConA (2.5 µg/mL) as controls of unspecific polyclonal stimulation.

### 2.10. Determination of Autoantibodies

ELISA was used to determine the specific anti-MOG_35–55_ antibody responses. Maxisorb plates (Millipore) were coated overnight at 4 °C with MOG_35–55_ (10 µg/mL) in 0.1 M biphosphate buffer (pH 9.6), blocked with PBS/10% FBS, and incubated for 2 h at 37 °C with serial dilutions of sera obtained by cardiac puncture at the disease peak. Biotin-labeled anti- IgG1 or anti-IgG2a/c antibodies (2.5 µg/mL; Serotec) were added for 1 h at 37 °C. After washing, plates were incubated with streptavidin-peroxidase HRP, developed with the HRP substrate ABTS, and absorbance was determined in a spectrophotometer.

### 2.11. Spinal Cord Organotypic Cultures and Excitotoxicity Assays

Lumbar spinal cords were aseptically obtained from 8-day-old Sprague-Dawley rat pups previously euthanized by pentobarbital overdose. The tissue was placed in ice-cold high glucose containing (6.4 mg/mL) Gey’s Balanced Salt Solution (GBSS). Meninges and roots were removed, and the spinal cord was transversely sectioned into 350 µm thick slices with a McIlwain Tissue Chopper. Four slices were carefully transferred onto 30-mm-diameter Millicell-CM membranes (0.4 µm, Millipore, Billerica, MA, USA) into a 6-well plate (35-mm-diameter, Falcon) containing 1 mL of incubation medium [50% (*v*/*v*) minimal essential medium (MEM), 25 mM Hepes, 25% (*v*/*v*) heat- inactivated horse serum, 2 mM glutamine and 25% (*v*/*v*) Hank’s Balanced Salt Solution (HBSS) supplemented with 25.6 mg/mL glucose; pH 7.2. Cultures were allowed to stabilize for 1 week at 37 °C in a 5% CO_2_/95% air humidified environment, and thereafter medium was changed twice per week. After 7 days of the explant culture, chronic glutamate excitotoxicity was induced by exposure at DL-threo-β-hydroxyaspartic acid (THA; 100 µM; Sigma-Aldrich, Saint Louis, MO, USA). Some slices were treated with ORY-2001 (at 5, 25 and 125 nM), and Riluzole (at 5 µM) was used as a positive control. THA and treated compounds were renewed at each medium exchange, twice per week until 28 days in vitro (DIV). In the different experimental conditions, slices were fixed with 4% paraformaldehyde in TBS for 1 h at room temperature. After blocking with 5% normal goat serum and 0.3% Triton-X-100 in TBS, the sections were incubated with a primary antibody against a anti-neurofilament heavy chain (SMI-32; BioLegend, San Diego, CA, USA; 1:1000) to label motoneurons. Cultures were then thoroughly washed in TBS with 0.1% Tween-20 (TBS-T) and incubated for 1 h at room temperature with donkey anti mouse IgG (H+L) highly cross-adsorbed secondary antibody (Invitrogen, Carlsbad, CA, USA; 1:1000). After several washes, sections were counterstained with DAPI and mounted in Fluoromount-G medium (Southern Biotech). Images of the ventral horn of each hemislice were captured with a scanning confocal microscope (LSM 700 Axio Observer, Carl Zeiss 20×/z0.5). Motoneurons, identified by SMI-32 immunostaining and localization in the ventral horn, were counted by using the Cell Counter plugin of ImageJ software. A minimum of 12 sections were evaluated for each experimental condition. Cord sections damaged during processing, which did not correspond to lumbar segments or without clear labeling were excluded from statistical analysis.

### 2.12. Analysis of Gene Expression

We evaluated differences in gene expression induced by the treatment using real-time PCR and by microarray hybridization analysis. First, total RNA was isolated from brain and spinal cord segments from EAE mice using Qiazol™ and the RNeasy MiniKit™ extraction kit (Qiagen). Evaluation of RNA quality was performed with the Agilent 2100 bioanalyzer and NanoDrop™ ND-1000 (Thermo Scientific, Dartford, Kent, UK). After DNAse I treatment, RNA (1 µg/sample) was reverse transcribed using RevertAid First strand cDNA Synthesis kit (Fermentas) and random hexamer primers. For evaluating the expression of the inflammatory markers *TNFα*, *IL-1β*, *IP10*, *IL-4*, and *CCL5*, real time PCR was performed by using iQ SYBR Green Supermix (Bio-Rad, Feldkirchen, Germany) according to manufacturer’s instructions in a BIORAD CFX96 thermal cycler. Primer sequences and PCR conditions are indicated in Supplementary Materials Table S3. Microarray studies were performed by using DNA microarray oligo probes designed with *Tethys*, Oryzon’s proprietary software which is based on the in silico thermodynamic simulation of hybridization. Agilent probes were added for genes which yielded no probes, complying with *Tethys* criteria. Control probes were used to assess detection limits and range, to verify spatial homogeneity, and to determine experimental within-array variation. Microarray slides were synthesized by Agilent. Total RNA (0.5 µg) amplification and labeling with Cy3 or Cy5 was carried out using a modified Eberwine mRNA amplification procedure [36,37] employing the MessageAmp™ II aRNA Amplification kit from Ambion (ThermoFisher Scientific, Oxford, UK). The Cy3- and Cy5-labelled cRNA mixes were hybridized to microarrays according to the manufacturer’s instruction. Raw data were obtained using Agilent’s DNA Microarray Scanner G2505B and Feature Extraction software (v10.1, Santa Clara, CA, USA) and processed using the proprietary *Polyphemus* software. Validation of microarray biomarkers by qRT-PCR was performed starting from 0.5 µg of total RNA with the High-Capacity RNA to cDNA Master Mix (Invitrogen) or iScript^®^ Reverse Transcription Supermix for qRT-PCR (Bio-Rad). qRT-PCR reactions were performed in triplicate using 10–20 ng of first strand DNA and off-the-shelf Taqman assays in a Roche Lightcycler II 480, according to the manufacturers’ recommendations. Taqman assays used were purchased from ThermoFisher Scientific and are included in Supplementary Materials Table S1. Expression of *Gapdh* was used for normalization, and fold change expression was estimated using the −ΔΔCt method.

### 2.13. Biochemical Assays

HDAC2 activity was determined using a fluorimetric assay at BPS Bioscience (San Diego, CA, USA). Compounds were dissolved in DMSO (Sigma), and serial dilutions were further diluted in HDAC assay buffer (BPS). These working solutions were incubated in duplicate at room temperature for 3 h in a mixture containing HDAC assay buffer, BSA (Fisher Scientific), and recombinant HDAC2 (BPS). The enzymatic reactions were initiated by the addition of a fluorogenic, acetylated peptide substrate (BPS) and proceeded for 30 min at 37 °C. Then, HDAC assay developer (BPS) was added, and after further incubation at room temperature, fluorescence intensity was measured at an excitation of 360 nm and an emission of 460 nm using a Tecan Infinite M1000 microplate reader. Trichostatin A (TSA, Selleckchem, Houston, TX, USA) was used as a reference inhibitor.

### 2.14. Cellular Assays

Cell on impedance modulation, using the CellKey (Cellular Dielectric Spectroscopy) detection method (CellKey 96, MDS Sciex, Concord, ON, Canada) at Eurofins Pharma Discovery Services (Celle-Lévescault, France). CHO cells were seeded onto a 96-well plate at 15.104 cells/well in HBSS buffer (Invitrogen), 20 mM HEPES (Invitrogen), 10% FCS (Invitrogen), and 0.1% BSA (Sigma), allowed to equilibrate for 60 min at 28 °C. DMSO solutions of compounds were diluted in HBSS and 0.1% BSA. Plates were placed onto the system, and measurements were made at a temperature of 28 °C. To measure the agonistic effect, compounds and controls in duplicate were added simultaneously to all of the wells using an integrated fluidics system. Impedance measurements were monitored for 10 min after ligand addition. S1P (Sigma) was used as reference agonist (EC_50_ = 0.001 µM). To measure the antagonistic effect, compounds and controls in duplicate were pre-incubated for 15 min before the addition of the reference agonist S1P at 0.01 µM (EC_80_). Impedance measurements were monitored for 10 min. VPC23019 (Avanti Polar Lipids) was used as a reference antagonist (IC_50_ = 0.027 µM).

Human H3 acetylation assays: for the determination of global and K9-specific acetylation levels, 500,000 SH-SY5Y cells were seeded in 6-well plates in DMEM/F12 1:1 (Sigma), supplemented with 2 mM glutamine (LabClinics) and 10% FBS (Sigma) and incubated at 37 °C and 5% CO_2_ in a humid atmosphere. SH-SY5Y cells were treated 24 h after seeding with SAHA (Selleckchem), Fingolimod (FTY720, Selleckchem), Fingolimod Phosphate (FTY720-P, Cayman Chemical Company) and ORY- 2001. After 2 and 6 h of treatment, cells were harvested and centrifuged at 250× *g* for 4 min at room temperature and cell pellets used for total histone extraction with EpiQuik Total Histone Extraction Kit (Epigentek, Farmingdale, NY, USA) following manufacturer’s instructions. 1 µg of histone extracts were run in MOPS buffer in 12% PAGE (Life Technologies, Carlsbad, CA, USA) and transferred with the iBlot system to nitrocellulose membranes. Transferred blots were stained with the Ponceau S solution (Sigma) and scanned with an Epson Perfection V600 Photo Scanner. Membranes were blocked in blocking buffer (5% skimmed milk in PBS-Tween 0.1%) for 1 h at room temperature, and after blocking blots were incubated with rabbit polyclonal anti-H3K9Ac (1:2000 dilution, Abcam, Cambridge, UK) and rabbit polyclonal anti-H3ac (K9 + K14 + K18 + K23 + K27) (1:2000 dilution, Abcam) overnight at 4 °C in ablocking buffer. Membranes were washed 3 *×* 5 mins with PBS- Tween 0.5% and incubated with peroxidase-conjugated donkey anti-rabbit IgG (1:10.000 dilution, Life Technologies) for 1 h at room temperature in a blocking buffer. After 3 washes with PBS-Tween 0.5% of 5 min each and 1 wash of PBS 1X, blots were developed with the ECL Prime Western Blotting Detection Reagent (Amersham, Leicestershire, UK) and imaged in the G:BOX XRQ imaging system (Syngene, Cambridge, UK).

### 2.15. Statistical Analysis

For animal models and cellular responses, all data are expressed as the mean ± SEM. N and *n* reflect the number of biological and technical replicas, respectively. Statistical analysis was carried out with two-way ANOVA followed by Bonferroni multicomparison test (for specific EAE daily scores) and by *t*-test and non-parametric Mann–Whitney test for the rest of EAE score comparisons. Dixon’s exclusion criteria were applied to TMEV raw data. Analysis via one-way ANOVA and followed by Tukey’s multiple comparisons test were performed for TMEV scores, EAE autoreactive responses, and flow cytometry results, as well as by one-way ANOVA and followed by Dunnett’s post hoc test for evaluating spinal cord explant results. 

For the gene expression studies, data from microarray assays were normalized by modified nonlinear Q-splines normalization method and Log2(Sample/Vehicle) values calculated without background correction (which permits robust selection of differentially expressed genes yet may lead to sub-estimation of the magnitude of change for genes expressed near the detection limit). Differential expression was assessed with the proprietary *Polyphemus* software using robust statistics on the average technical replicates (3 replicates/gene oligo datapoint) after removing eventual outlier points (caused by dust or array imperfections). *Polyphemous* automatically defines the criteria for outlier elimination by assessing the intra-array technical variability using the signal distribution of controls probes (a large number of replicates present on the array). The *p* values were calculated after outlier elimination based on the absolute value of the regularized t-statistics, which uses a Bayesian framework to derive the algorithm, using internal replicated controls to assess the minimum technical variability of the process. Correlation between gene expression changes induced by different treatments was analysed by calculating the Pearson correlation coefficient r using the Log2(Treatment/Veh) values for all genes expressed above background level (n). The t values for the student’s t test were calculated as: r × (df)1/2 × (1 – r2) – ½; with the degrees of freedom df = n – 2. The *p* values were calculated as 2 × tcd(t,df) using the t distribution function with the Keisan Online Calculator service (https://keisan.casio.com/calculator). The analysis is highly sensitive and efficiently detects small systematic biases in the data measurement system, i.e., a difference in labeling efficiency in the Cy3 and Cy5 channel. Nevertheless, the *p* values for the correlations between the Log2(Treatment/Veh) values for each treatment were much lower than the *p* values for the correlations between the Log2(Treatment/Veh) and Log2(Veh/Veh) values (Supplementary Materials Table S2). In the real-time analysis, mean Cp values for each datapoint were calculated after outlier elimination using Grubbs test (applied if the standard deviation of three technical PCR replicates was higher than Cp 0.25). −ΔΔCp (or analogous −ΔΔCt) values relative to Vehicle were calculated as follows: −([Cp gene − Cp endogenous]_Treatment_-average{[Cp gene − Cp endogenous]vehicle}). Vehicle and ORY-2001 were compared by unpaired *t*-test. Welch’s correction was applied when the two distributions showed unequal variances.

For the biochemical assays, the fluorescence signal relative to the background (assay mixture in the absence of the enzyme) was subtracted from each fluorescent intensity data. Activity in the presence of compound was expressed as a percentage of fluorescence intensity in the absence of the compound.

We considered significance to be at *p* < 0.05. All statistical analyses were performed using the Graph-Pad Prism software v8.3.0, San Diego, CA, USA.

## 3. Results

### 3.1. Treatment with ORY-2001 Protects from Chronic EAE

We first investigated the effect of the oral administration of KDM1A inhibitors in the chronic progressive EAE model induced by MOG_35–55_ in C57BL/6 mice (Figure 1a). The disease severity in the model has been found to present seasonal variations [34], but generally, all mice develop clinical signs and do not recover from the disease. 

In the first experiment, ORY-2001 and the selective KDM1A inhibitor ORY-LSD1 were tested. The compounds have different potencies, but doses with an equivalent in vivo impact in MTD studies were used (1 mg/kg for ORY-2001 and 180 µg/kg for ORY-LSD1). Mice were treated for two weeks starting at the onset of disease at day 12 and were observed until day 51 post-immunization. The vehicle treated group displayed severe EAE (mean maximal clinical score ≈ 4.1). 40% of vehicle-treated EAE mice developed moderate (maximal clinical score of 2.0–2.5) and 60% severe signs (maximal clinical score 3.5–6), and mice did not recover from the disease (Supplementary Figure S1a). 

Treatment with ORY-2001 improved the clinical signs (Supplementary Figure S1a) and significantly reduced disease severity with a mean maximum clinical score of 1.8 (Supplementary Figure S1b,c). Only 10% of animals treated with ORY-2001 developed severe manifestations of the disease, and 60% showed mild clinical signs (clinical score ≤ 2). Treatment with the highest dose of ORY-LSD1 partially ameliorated the clinical signs but did not significantly reduce disease severity (Supplementary Figure S1a–c). The ORY-LSD1 group displayed a mean maximum clinical score of 3.0 at 180 µg/kg) and 40% of ORY-LSD1-treated animals exhibited severe signs of the disease (maximum clinical score ≥ 4.0) (Supplementary Figure S1c). 

In the second experiment, doses of ORY-2001 were lowered to assess the therapeutic window (0.05, 0.5 and 1 mg/kg), and the selective KDM1A inhibitor ORY-LSD1 (90 and 180 µg/kg) and MAO-B inhibitor rasagiline (3 mg/kg, sufficient for full inhibition in vivo) were included for comparison to assess the contribution of KDM1A and MAO-B inhibition in the therapeutic mechanism. The vehicle treated group displayed mild signs (mean clinical score ≈ 2, Figure 1). 55% of vehicles treated EAE mice developed moderate (maximum clinical score of 1.5–3) and 27% severe signs (maximum clinical score of 3.5–6). 

ORY-2001 improved clinical signs (Figure 1b) and significantly reduced disease severity (Figure 1e,f). Most mice treated with 0.5 or 1 mg/kg ORY-2001 displayed mild (50%) or no (10%) clinical signs. Notably, short term treatment with ORY-2001 was sufficient to generate a long-lasting protective effect, as a significant number of mice were almost asymptomatic (clinical score ≤ 0.5) 40 days after disease onset. Only the group treated with the lowest dose (0.05 mg/kg) suffered a relapse during the last week of follow-up (Figure 1b), reflecting a partial loss of efficacy. The therapeutic window found in the EAE model (corrected for the dosage form) was similar to that observed previously in the novel object recognition test in SAMP8 mice, shown to correlate with KDM1A target engagement [20]. Importantly, treatment with ORY-2001 was effective at doses that did not significantly impact the total number of circulating lymphocytes in toxicity studies (Table S1, in Supplementary Material).

ORY-LSD1 partially ameliorated the clinical signs (Figure 1c) but did not significantly reduce disease severity (Figure 1e,f). Treatment with rasagiline seemed to induce a slight delay of the onset of the clinical signs (Figure 1d) but showed no overall protective effect on the clinical course or the severity of the disease (Figure 1e,f). At the highest dose tested, the maximum clinical scores registered for the individual mice reflected none of the ORY-2001, 10% of ORY-LSD1, 33% of rasagiline, and 30% of vehicle-treated animals exhibited severe signs (maximum clinical score 3.5–6) in this experiment (Figure 1f).

Interestingly, at the end of the study, 70–80% of EAE mice treated with ORY-2001 and 60% of ORY-LSD1-treated mice either did not show any or showed only mild clinical signs, whereas only 44% of the rasagiline and 33% of vehicle-treated mice displayed such a phenotype.

Together, these results show that the inhibition of KDM1A but not MAO-B is key to the therapeutic effect of ORY-2001 in the EAE model.

### 3.2. ORY-2001 Reduces Inflammatory Infiltration and Demyelination in EAE Mice

**Figure 2:**
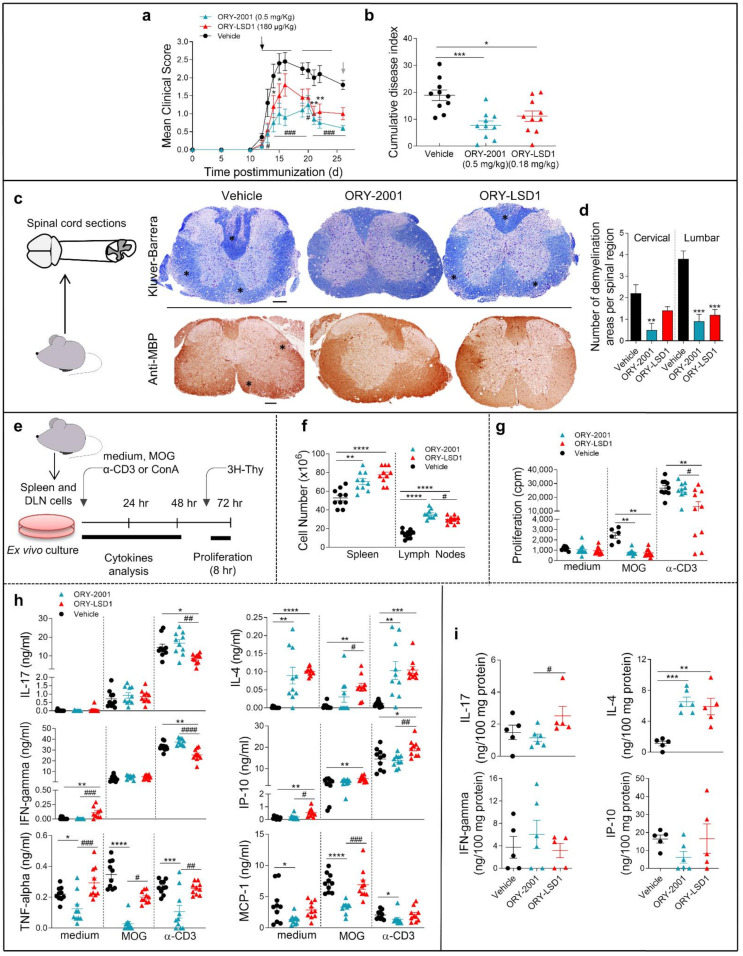
ORY-2001 protects from demyelination and inflammation in the EAE mice by modulating the immune infiltration to the CNS. Mice with MOG_35–55_-induced chronic EAE were orally treated with vehicle, ORY-2001 (0.5 mg/kg) or ORY-LSD1 (180 µg/kg) for two periods of five days (horizontal bars in (**a**)) starting at the onset on day 12 (pointed by a black arrow, (**a**)) Mean clinical score (**a**) and cumulative disease index (**b**) are showed for each group until animals were sacrificed (pointed by a grey arrow 26 days after immunization, sub-chronic phase). Data represent mean ± SEM. N = 10 mice/group; (**a**) * *p* < 0.05, ** *p* < 0.01 between vehicle and ORY-LSD1-treated mice; # *p* < 0.05, ### *p* < 0.0005 between vehicle and ORY-LSD1-treated mice; (**b**) * *p* < 0.05, *** *p* < 0.0005 between vehicle and treated mice. (**c**) Spinal cords were isolated at the end of the treatment (26 days post-immunization) and processed for histopathological analysis of the disease. Transverse sections of cervical spinal cord randomly selected in each group were stained with Klüver-Barrera (upper images) or immunostained for myelin content with anti-MBP (lower images). Stars point to areas of demyelination and inflammatory infiltration. Images are representative of 5 mice/group. Scale bars: 200 µm; (**d**) The mean number of plaques of demyelination in the lumbar and cervical regions was determined. ** *p* < 0.01, *** *p* < 0.0005 between vehicle and treated mice; (**e**) Schematic representation for evaluating cell content and autoreactive responses by spleen cells in (**f**–**h**) isolated at sub-chronic EAE (26 days post-immunization) and stimulated with medium, the encephalitogenic Ag (MOG35-55), or a polyclonal stimulus (anti-CD3 Ab). Cell number in spleen and lymph nodes; (**f**) proliferation (thymidine-incorporation); (**g**) and cytokine/chemokine production of spleen cells; (**h**) was determined from vehicle, ORY-2001 and ORY-LSD1-treated EAE mice at the sub-chronic phase. N = 7–10 mice/group. * *p* < 0.05, ** *p* < 0.01, *** *p* < 0.0005, **** *p* < 0.0001 between vehicle and treated mice; # *p* < 0.05, ## *p* < 0.01, ### *p* < 0.0005, #### *p* < 0.0001 between ORY-2001 and ORY-LSD1-EAE treated mice; (**i**) Levels of inflammatory mediators determined by ELISA in protein extracts purified from spinal cord at the sub-chronic phase, N = 5–6 mice/group. ** *p* < 0.01, *** *p* < 0.0005 between vehicle and treated mice; # *p* < 0.05 between ORY-2001 and ORY-LSD1-EAE treated mice. Data are the mean ± SEM with dots represents individual values of biologically independent animals.

To elucidate the mechanism of action of ORY-2001 and ORY-LSD1, we performed an EAE experiment in which animals were sacrificed in the sub-chronic phase of the disease on day 26 (Figure 2). As expected, at the peak of the disease, while all the vehicle-treated animals developed moderate signs of EAE, both compounds reduced the clinical signs (Figure 2a,b). However, ORY-2001 showed more potent protective effects as all the ORY-2001-treated mice displayed only mild symptoms while 30% of the ORY-LSD1-treated animals displayed a moderate clinical score. Next, we investigated focal areas of inflammatory infiltration and demyelination, two main features of the spinal cord pathology in MS and EAE. A histopathologic examination of the spinal cords of vehicle-treated EAE mice showed many plaques in both lumbar and cervical segments (Figure 2c,d, Supplementary Figure S2). Notably, treatment with ORY-2001 significantly reduced the number of plaques with inflammatory infiltrates and areas of demyelination (Figure 2c,d, Supplementary Figure S2), which could explain the protective effect observed for this compound and the avoidance of the progression of the disease to a severe state in EAE. Although treatment with ORY-LSD1 also resulted in a remarkable decrease in the number and size of demyelinating plaques and inflammatory foci in the spinal cord, this effect was less prominent than that exerted by ORY-2001, mainly in the cervical areas (Figure 2c,d, Supplementary Figure S2). In EAE and MS, CNS immune infiltration and demyelination is correlated with the activation of the peripheral autoreactive IFNγ-producing Th1 and IL-17-secreting Th17 cells. Therefore, in order to investigate if the KDM1A inhibitors could ameliorate EAE by modulating encephalitogenic T-cell responses and/or their migration to CNS, we analyzed the inflammatory and autoimmune response in the peripheral immune organs (Figure 2e). Interestingly, we found a significant increase in the number of immune cells in the spleen and lymph nodes of the animals treated with ORY-2001 and ORY-LSD1 (Figure 2f). This suggests that both compounds could be affecting the lymphocyte migration to the CNS by inducing their accumulation in the peripheral lymphoid organs and, indirectly, by avoiding their recruitment to the target tissues in the CNS. Spleen lymphocytes isolated from ORY-2001 treated mice showed reduced MOG_35–55_ but not anti-CD3 -stimulated proliferation, while lymphocytes from the spleen of ORY-LSD1-treated mice showed both reduced MOG_35–55_ and reduced anti-CD3-stimulated proliferation, compared to those from vehicle-treated animals (Figure 2g).

Although T cells isolated from ORY-2001 or ORY-LSD1-treated mice produced equivalent levels of IFNγ, IL-17, and IP-10 in the MOG_35–55_ -specific recall response, only the treatment with ORY-2001 significantly reduced the levels of TNF-alpha and MCP-1. On the other hand, the production of the anti-inflammatory cytokine IL-4 was increased with both treatments (Figure 2h). 

The effects observed were partially antigen-specific as both compounds affect polyclonal T cell activation with anti-CD3 antibody and the reactivation responses with the myelin antigen in a similar manner. 

**Figure 3:**
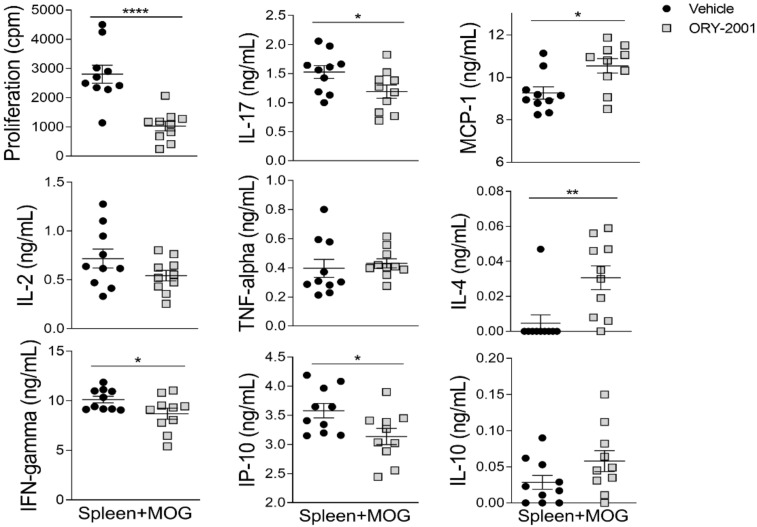
Treatment with ORY-2001 deactivates encephalitogenic T-cell responses in vitro. Proliferative response and cytokine/chemokine production by spleen cells isolated from mice suffering chronic EAE at the peak of the disease and then re-stimulated with MOG_35–55_ (15 µM) in the absence (vehicle) or presence of 0.5 µM ORY-2001 (i.e., 5 *×* IC_50_ for KDM1A, sufficient for full inhibition). N = 10/group. * *p* < 0.05, ** *p* < 0.01, **** *p* < 0.0001 between vehicle and ORY-2001. Data are the mean ± SEM with dots representing individual values of biologically independent animals.

As the activation of infiltrating cells leads to de-regulation of immune processes of resident glial cells in MS and EAE, we also analyzed the levels of inflammatory factors in the spinal cord, which is the main target tissue in EAE. Similar to the results in the periphery (Figure 2h), no significant changes were found in the levels of IFNγ, IL-17, and IP-10 in the spinal cord of vehicle, ORY-2001, and ORY-LSD1-treated EAE mice (Figure 2i). However, a significant increase in the levels of IL-4 can be observed in the spinal cords of animals treated with ORY-2001 and ORY-LSD1 (Figure 2i). These results indicate that a direct effect on Th1/Th17 cell-driven encephalitogenic response in lymphoid organs is not likely to be involved in the protective effect of ORY-2001 and ORY-LSD1 in chronic EAE (at least at the time point assayed). Although we found similar results with ORY-2001 and ORY-LSD1 in some of the evaluated mediators (as the induction of IL-4-producing Th2 cells), the higher significant reduction of TNF-alpha and MCP-1 after the treatment with ORY-2001 could contribute to its improved protective response versus ORY-LSD1. In order to evaluate whether the effect of ORY-2001 is exerted directly on lymphoid cells, the exogenous addition of this compound to encephalitogenic T-cell cultures was evaluated. ORY-2001 deactivated the MOG_35–55_ -induced proliferation and cytokine production in vitro, while it induced a Th2 response by increasing the levels of the anti-inflammatory mediators IL-10 and IL-4 (Figure 3).

### 3.3. Treatment with ORY-2001 Improves Motor Function and Reduces the Clinical Score in the TMEV Model of Multiple Sclerosis

**Figure 4:**
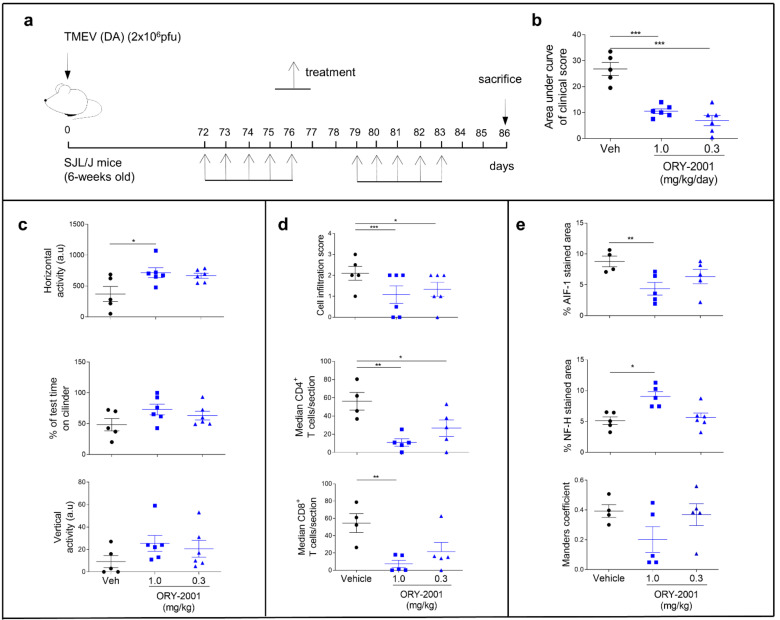
Therapeutic effects of ORY-2001 in the Theiler model. (**a**) Susceptible mice were infected with Daniel’s strain (DA) of TMEV into the cerebral parenchyma and treated orally with ORY-2001 (two different concentrations: 1.0 and 3.0 mg/kg) for two weeks following the onset of symptoms (72 days post-infection); (**b**) Clinical score was evaluated as the area under curve; (**c**) Motor function was analyzed by the horizontal and vertical activity (HACTV and VACTV, respectively) in the activity cage test and coordination through the rotarod test. Histopathological analyses were performed to examine immune infiltration (**d**) and neuroinflammation (**e**); (**d**) Immune cell infiltration score (upper graph) was determined, and the specific CD^4+^ and CD^8+^ T cells (medium and lower graph, respectively) were quantified in randomly selected sections of spinal cords from vehicle and ORY-2001-treated TMEV-infected mice; (**e**) Microglial activation analysis (upper graph, represented by the mean of the percentage of AIF-1 stained area), axonal integrity (medium graph, represented by the mean of the percentage of Neurofilament H positive area), and astroglial activation (lower graph, represents the mean of the Manders coefficient between Vimentin and GFAP staining) were performed on 6–12 images/mice from the ventral horn of spinal cord and data represents the mean of the percentage of respective staining in the cervical and thoracic regions of each animal. N = 4–6 mice/group; *n* = 4–6 spinal cord sections/mice. * *p* < 0.05, ** *p* < 0.01, *** *p* < 0.0005 between vehicle and ORY-2001. Data are the mean ± SEM with dots representing individual values of biologically independent animals.

To further confirm the potential of ORY-2001 for the treatment of MS, we tested ORY-2001 in the second established preclinical model for the disease: the Theiler’s murine encephalomyelitis virus (TMEV) model. In this model, susceptible mice infected with Daniel’s strain of TMEV develop a late-onset demyelinating disease which is pathologically similar to human MS. Oral treatment with different concentrations of ORY-2001 (during two weeks following onset of symptoms, Figure 4a) significantly improved symptomatology associated with intracranial TMEV infection and reduced the clinical score (Figure 4b), while it did not produce any body weight changes. As expected, TMEV infection affected horizontal and vertical activity and the performance in the rotarod test (Figure 4c). Treatment with ORY-2001 significantly improved the motor activity in the horizontal activity and partially reduced other motor deficits exerted by vehicle-treated TMEV (Figure 4c). On the other hand, the infiltration of immune cells into the spinal cord, increased by TMEV infection, was diminished after ORY-2001 treatment, which specifically decreased the accumulation of CD4 and CD8 lymphocytes into the parenchyma (Figure 4d). Furthermore, ORY-2001 significantly reduced microglial activation (AIF-1 positive cells), increased axon integrity, and partially limited astroglial activation in the spinal cord of TMEV mice (Figure 4e). These results indicate that ORY-2001 may restrict the migration of immune cells into the CNS, diminishing the neuroinflammation and axonal loss and leading to the potential effect of ORY-2001 in the restoration of the motor function in TMEV-infected mice.

### 3.4. ORY-2001 Protects Motoneurons from Chronic Excitotoxic Stress

**Figure 5:**
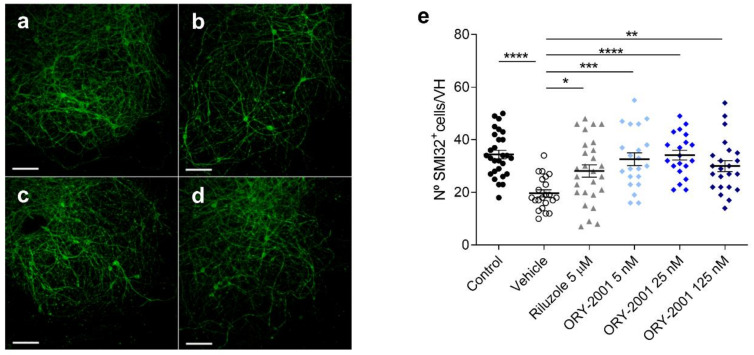
The effect of ORY-2001 on motoneurons injured by chronic excitotoxicity. (**a**–**d**) SMI-32 immunohistochemistry of (**a**) control spinal cord explants and spinal cord explants exposed to THA treated with (**b**) vehicle, (**c**) 5 μM riluzole or (**d**) 5 nM ORY-2001 in a chronic excitotoxicity assay. Scale bar: 100 μm. (**e**) Quantification of SMI-32+ motoneurons in control spinal cord explants (*n* = 28) and explants exposed to THA treated with vehicle (*n* = 22 hemislices), 5 μM riluzole (*n* = 27 hemislices) or with ORY-2001 5 (*n* = 21 hemislices), 25 (*n* = 20 hemislices) or 125 nM (*n* = 23 hemislices). Data are pooled from 3 experiments performed on different days and presented as mean ± SEM. * *p* < 0.05, ** *p* < 0.01, *** *p* < 0.001, **** *p* < 0.0001.

After evaluating immunomodulatory actions of ORY-2001, we sought to evaluate the neuroprotective effect for this compound (partially suggested in the results with TMEV model). In MS, glutamate (Glu)-induced excitotoxicity has been proposed to constitute a pathogenic mechanism of neuronal injury [37,38]. Threo-β-hydroxyaspartate (THA) induced organotypic spinal cord explants are used as a model of chronic excitotoxicity: THA blocks glutamate transporters, provoking a toxic increase of glutamate and intraneuronal Ca(^2+^) and leading to around 40% motoneuron death after three and 80% cell death after for weeks of treatment. This chronic model has been deemed appropriate to study epigenetic modulators [39]. To evaluate the neuroprotective effect of ORY-2001, we studied the effect of different concentrations of this compound on the survival of SMI32 positive motoneurons in explants challenged with THA. We observed that ORY-2001 protected the motoneurons from chronic excitotoxic stress at all doses tested and outperformed the effect exerted by riluzole, used as a positive control (Figure 5a–e).

### 3.5. Comparison of the Effects Exerted by ORY-2001 and Current Oral Drugs for the Treatment of MS

**Figure 6:**
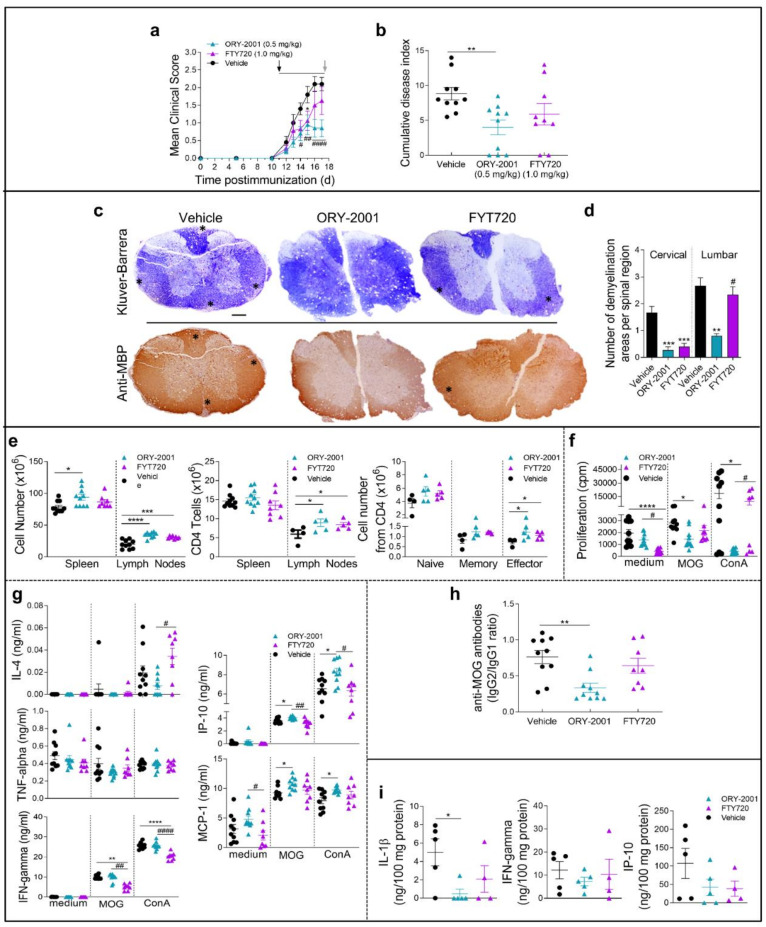
A comparison of the effects of ORY-2001 and FTY720 in EAE mice. Mice with MOG_35–55_-induced chronic EAE were orally treated with vehicle, ORY-2001 (0.5 mg/kg) or FTY720 (1.0 mg/kg) for five consecutive days (horizontal bar in a) starting at the onset of EAE on day 12 (pointed by a black arrow, (**a**)). Mean clinical score (**a**) and cumulative disease index (**b**) are showed for each group until animals were sacrificed 24 h after last treatment (corresponding to the peak of the disease, effector phase, pointed by a grey arrow, (**a**)). Data represent mean ± SEM. N = 10 mice/group; (**a**) * *p* < 0.05 between vehicle and FTY720-treated mice; # *p* < 0.05, ## *p* < 0.01, #### *p* < 0.0001 between vehicle and ORY-LSD1-treated mice; (**b**) ** *p* < 0.01 between vehicle and ORY-2001-treated mice; (**c**) Transverse sections of cervical spinal cord randomly selected at the peak of chronic mild-EAE (17 days post-immunization) stained with Klüver-Barrera (upper images) and for myelin content (anti-MBP, lower images) to detect areas of demyelination and inflammatory infiltration (stars). Images are representative of 5 mice/group. Scale bars: 200 µm; (**d**) The mean number of plaques of demyelination in the lumbar and cervical regions was determined. ** *p* < 0.01, *** *p* < 0.0005 between vehicle and treated mice; # *p* < 0.05 between ORY-2001 and FTY720-treated mice; (**e**) Total number of cells was quantified in dissected and homogenized lymph nodes and spleen isolated at the peak of EAE from mice treated with vehicle, ORY-2001 and FTY720. CD4 T cells were determined and lineage expression in the CD4 population was assayed by flow cytometry (CD4/CD44^low^/CD62L^high^ as naïve T cells; CD4/CD44^high^/CD62L^low^ as memory T cells; CD4/CD44^medium^/CD62L^low^ as effector T cells). N = 4–10 mice/group. * *p* < 0.05, *** *p* < 0.001, **** *p* < 0.0001 between vehicle and treated mice. Proliferation; (**f**) and cytokine/chemokine production by spleen cells isolated at the disease peak and incubated with medium or stimulated with the encephalitogenic Ag (MOG_35–55_), or a polyclonal stimulus (ConA); (**g**). N = 8–10 mice/group. * *p* < 0.05, ** *p* < 0.01, **** *p* < 0.0001 between vehicle and treated mice; # *p* < 0.05, #### *p* < 0.0001 between ORY-2001 and ORY-LSD1-EAE treated mice; (**h**) MOG_35–55_-specific IgG1 and IgG2 levels in sera collected at the disease peak (*n* = 8–10 mice/group). ** *p* < 0.01 between vehicle and ORY-2001-treated mice; (**i**) Levels of inflammatory cytokines and chemokines were determined by ELISA in protein extracts purified from spinal cord at the peak of the disease. N = 4–5 mice/group. * *p* < 0.05 between vehicle and ORY-2001-treated mice. Data are the mean ± SEM with dots representing individual values of biologically independent animals.

Our results have shown that the modulation of cellular migration from the periphery to the CNS is a key component of the mechanism of action of ORY-2001 in mouse models for chronic MS. The reduction of lymphocyte egress is a hallmark of the mechanism of drugs targeting the Sphingosine 1 phosphate receptors (S1PRs) like fingolimod (FTY720), an oral drug currently used for the treatment of relapsing-remitting multiple sclerosis [40]. We performed a side-by-side comparison of the effects of ORY-2001 and FTY720 in the effector phase of the EAE mice model and animals were sacrificed at the time of the expected maximal clinical score (Figure 6). Vehicle treated animals developed signs of mild EAE (mean clinical score 2.1). As expected, oral treatment with ORY-2001 and FTY720 initiated at the time of onset reduced the mean clinical score (Figure 6a), although only ORY-2001 significantly decreased the cumulative disease index compared to vehicle-treated EAE mice (Figure 6b). A histopathological analysis of target tissue revealed that while ORY-2001 reduced the presence of infiltrating/demyelinating plaques in both cervical and lumbar segments, FTY720 was less effective and reduced the number and size of demyelinating plaques in cervical but not in lumbar sections of the spinal cord (Figure 6c,d, Supplementary Figure S3). Both compounds increased the cellularity in lymph nodes although only ORY-2001 increased it significantly in the spleen (Figure 6e). Treatment with both drugs increased the CD^4+^ T cell population of the lymph nodes, and while only a slight increase of naïve/memory cells was observed in this fraction, the number of effector T cells was significantly increased in comparison with the vehicle-treated group (Figure 6e). Interestingly, at the time point selected for evaluation, only ORY-2001 significantly reduced the MOG_35–55_-induced proliferation of spleen cells, reflecting a reduced autoreactive response (Figure 6f).

On the other hand, while only FTY720 reduced the levels of IFNγ and increased IL-4 release in Con A-stimulated spleen cells, the treatment with ORY-2001 neither affected such cytokines (at the time of study) nor TNF-alpha. Of note, only the treatment with ORY-2001 resulted in a significant increase in the production of the pro-inflammatory chemokines IP-10 and MCP-1 induced by MOG_35–55_ (Figure 6g). Apparently, the effects observed for both compounds were partially antigen-specific because they were both affected in a similar way to that of the polyclonal T-cell activation with ConA and the recall responses with the myelin antigen. 

High levels of circulating antibodies against myelin antigens have been described to accompany the development of MS and EAE and to determine susceptibility to the disease. We examined the effect of ORY-2001 and FTY720 on the serum levels of MOG_35–55_-specific autoantibodies. We found that only the treatment with ORY-2001 significantly reduced the ratio between IgG2 (characteristic of Th1 responses) and IgG1 isotypes (indicative of Th2 functions) (Figure 6h). Finally, the levels of inflammatory mediators such as IFNγ and IP-10 were decreased in the spinal cord after the treatment with ORY-2001 and FTY720, and although this decrease did not reach significance, ORY-2001 significantly reduced the exacerbated levels of IL-1β found in vehicle-treated EAE mice and FTY720 showed the same tendency (Figure 6i). These results indicate that, as previously shown for samples obtained at the sub-chronic phase of the disease, treatment with ORY-2001 does not modify the autoreactive potential of T cells but seems to induce their accumulation in immune tissues, indirectly avoiding their recruitment to target tissues in the CNS. In general, although FTY720 showed similar effects, the treatment with ORY-2001 resulted in a higher decrease of the clinical symptoms of the EAE model evaluated at the effector phase probably corresponding to a stronger or faster regulation of T cell migration and a significant modulation of the production of autoantibodies by ORY-2001 that was not exerted by FTY720 at the chosen timepoint.

### 3.6. Gene Expression Regulation by ORY-2001, ORY-LSD1 and FTY720

**Figure 7:**
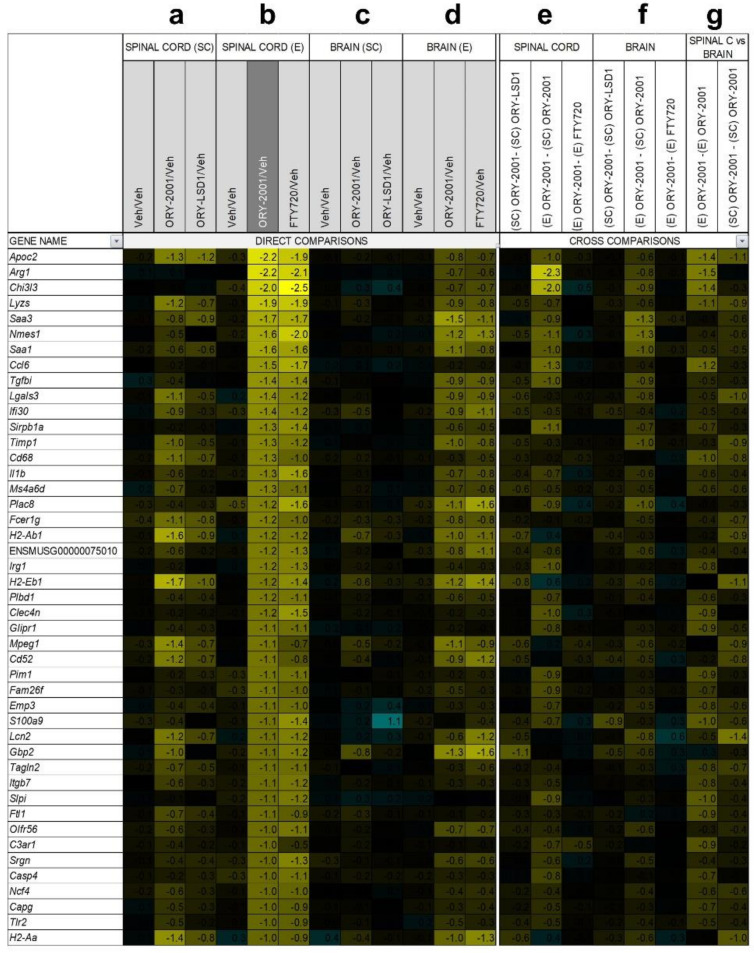
Genes down-regulated by ORY-2001. Microarray survey of gene expression changes in the spinal cord (**a**) and in the brain (**c**) in the sub-chronic phase (SC) 3 days after the last dose. Animals were treated with vehicle (Veh), ORY-2001 (0.5 mg/kg) or ORY-LSD1 (180 µg/kg). Microarray survey of gene expression changes in spinal cord (**b**) and in the brain (**d**) in the effector phase (**e**) 24 h after treatment. Animals were treated with vehicle, ORY-2001 (0.5 mg/kg) or FTY720 (1.0 mg/kg). Cross comparison of the effect of the different treatments and disease phases in the spinal cord (**e**) and in the brain (**f**); (**g**) Cross comparison of the effects of ORY-2001 in spinal cord and brain, in both phases. Pooled RNA from N = 5 spinal cords, 10 brains of vehicle, ORY-2001 or ORY-LSD1-treated mice or from N = 4 spinal cords, 6 brains of FTY720-treated mice was used to perform each survey, *n* = 3 replicate probes within an array. Gene expression changes are expressed as Log_2_(Treatment/Veh) for direct comparisons, and as the difference of the Log_2_(Treatment/Veh) values for the respective conditions in the cross-comparisons. Genes down-regulated > 2-fold (Log_2_(ORY-2001/Veh) < −1) by ORY-2001 in the spinal cord in the effector phase were selected and represented for all comparisons.

**Figure 8:**
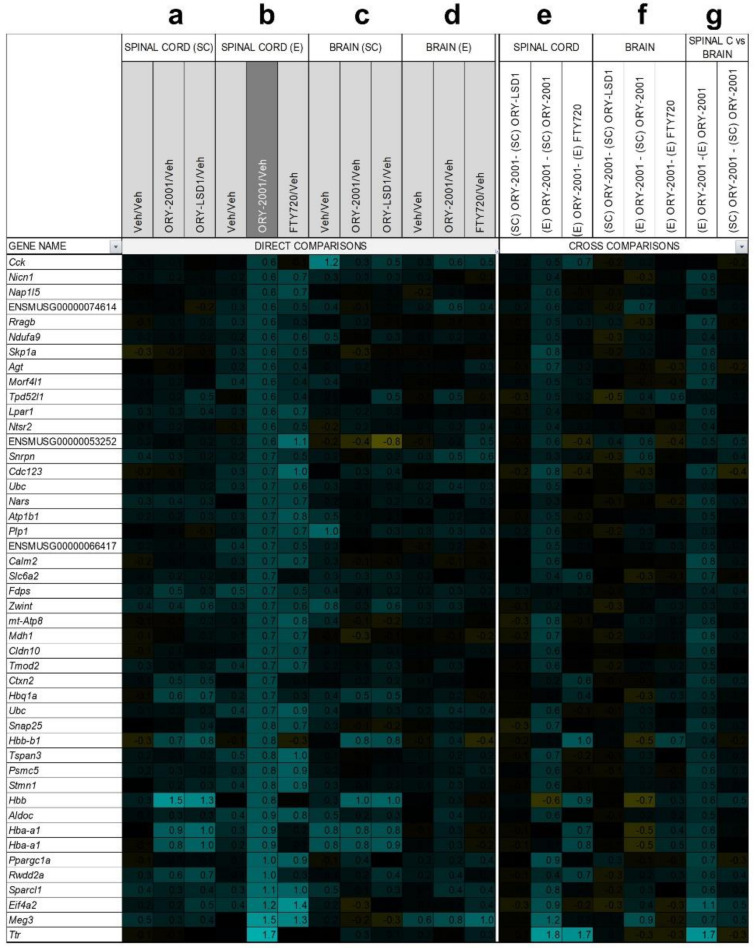
Genes up-regulated by ORY-2001. Microarray survey of gene expression changes in the spinal cord (**a**) and in the brain (**c**) in the sub-chronic phase (SC) 3 days after the last dose. Animals were treated with vehicle (Veh), ORY-2001 (0.5 mg/kg) or ORY-LSD1 (180 µg/kg). Microarray survey of gene expression changes in spinal cord (**b**) and in the brain (**d**) in the effector phase (**e**) 24 h after treatment. Animals were treated with vehicle, ORY-2001 (0.5 mg/kg) or FTY720 (1.0 mg/kg). Cross comparison of the effect of the different treatments and disease phases in the spinal cord (**e**) and in the brain (**f**). (**g**) Cross comparison of the effects of ORY-2001 in spinal cord and brain, in both phases. The number of samples for each survey, representation of changes in gene expression, and selected genes were performed as described in Figure 7.

Next, we decided to compare the anti-inflammatory profile of the compounds used in this study by performing a microarray-based gene expression surveys on pooled RNA samples from spinal cords and brains of vehicle and compounds-treated EAE mice (Figure 7 and Figure 8). First, in the spinal cord, by comparing ORY-2001 and ORY-LSD1 in the sub-chronic phase and in line with the therapeutic efficacy demonstrated for both compounds, we observed that changes in gene expression were more prominent for ORY-2001 (Figure 7a and Figure 8a and Supplementary Figure S4a). Among the top genes downregulated by ORY-2001, we found many genes described previously to be induced in EAE, such as those involved in antigen presentation (*H2-Eb1*, *H2-Aa*, *A2m*, *B2m* and others), complement factors (*C3*, *C1qa*, *C1qb*, *C1qc*), cytokines/chemokines (*Ccl5*, *Ccl8*, *Il1b*), microglial markers (*Aif1*, *Cd68*), factors involved in the breakdown of the blood brain barrier (*Lgals3*), modulators of demyelination/remyelination (*Cst7*, *Lcn2*, *Mpz*) or neuroprotection (*Wisp2*), in addition to drug targets for treatment of MS or related genes (*Cd52*, *Itgax*) (Figure 7a). On the other end of the spectrum, upregulated genes by ORY-2001 included *Cox1*, hemoglobin chain genes, mTOR signaling factors (*Ddit4*, *Dpysl2*), ubiquitin pathway genes (*Uchl1 and Rnf144*) and *Pou5f1*, a key factor for oligodendrocyte differentiation (Figure 8a). The inflammation in EAE transitions from a virulent effector phase to a more tempered chronic phase. Many of the inflammatory related genes upregulated in EAE and downregulated in the spinal cord after ORY-2001 treatment in the sub-chronic phase were modulated even stronger during the early effector phase (Figure 7b–e and Figure 8b–e and Supplementary Figure S4b–e); e.g., *Saa1*, *Nmes1 and Tgfbi*. On the contrary, inflammatory related genes including *Ccl6*, *Sirpb1*, *Irg1*, *Arg1*, *Chi3l3*, and *Ms4a8a*, selectively induced during the early phase of symptomatic development in EAE mice [41], were potently downregulated by ORY-2001 but exclusively in the effector phase. 

**Figure 9:**
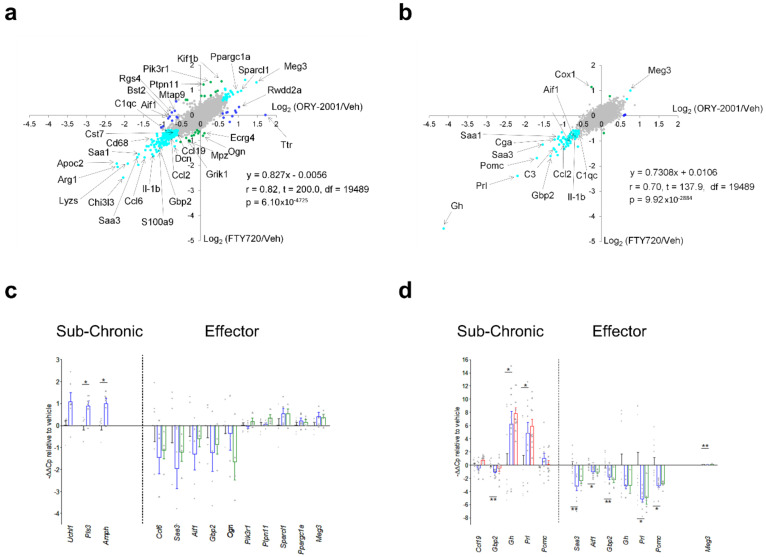
Comparisons of gene expression changes induced by ORY-2001, FTY720, and ORY-LSD1. Genome wide comparisons of expression changes induced by ORY-2001 and FTY720 in the spinal cord (**a**) or the brain (**b**) at the effector phase. Data are represented as scatterplots of Log_2_(ORY-2001/Veh) versus the Log_2_(FTY720/Veh) values. Genes regulated > 0.6-fold are represented in blue if they are specific to ORY-2001, in green if they are specific to FTY720, or in cyan if they are regulated in the same direction by ORY-2001 and FTY720. Microarray surveys were performed on pooled samples: (**a**) N = 5 mice/group (vehicle, ORY-2001), N = 4 (FTY720); (**b**) N = 10 mice/group (vehicle, ORY-2001), N = 6 (FTY720), and each datapoint was generated from a triplicate on array-measurement. Linear regression analysis calculated on 19,491 data points (**a**,**b**). r = Pearson correlation coefficient, t = t-Student test t value, df = degrees of freedom, *p* = probability of the two-tailed t-distribution for t and df. qRT-PCR validations of selected individual genes modulated by ORY-2001 (blue), ORY-LSD1 (red) and FTY720 (green) in the effector phase or sub-chronic phase in (**c**) spinal cord and (**d**) brain. Data are represented as −ΔΔCp values relative to vehicle and as mean ± SEM. In (**c**), samples for sub-chronic phase: N = 5 mice/group (vehicle), N = 6 mice/group (ORY-2001); effector phase: N = 5 mice/group (vehicle, ORY-2001), N = 4 mice/group (FTY720). In (**d**) samples for sub-chronic phase: *n* = 10 mice/group (vehicle, ORY-2001, ORY-LSD1); effector phase: *n* = 10 mice/group (vehicle, ORY-2001), N = 6 mice/group (FTY720). (**c**,**d**) For each mouse sample, the average of three technical replicate PCRs was calculated and is shown as a grey dot. Statistical analysis between vehicle and ORY-2001 was calculated using the two-tailed unpaired *t*-test. Welch’s correction was applied when the populations had unequal variances. * *p* < 0.05, ** *p* < 0.01.

Changes in gene expression induced in the spinal cord by ORY-2001 and FTY720 in the effector phase (Figure 7b and Figure 8b), were strongly correlated (r = 0.82) but more potent for ORY-2001, as can be deduced by the slope of the regression line reflected in the equation in Figure 9a (slope = 0.827) and *p* values (Supplementary Materials Table S2). *Apoc2*, *Arg1*, *Lyzs*, *Saa3*, *Nmes1*, *Saa1*, *Ccl6*, *Tgfbi*, *Lgals3*, *Sirpb1*, *Timp1*, *Cd68*, *Il1b*, *Ms4a6d*, *Fcer1g*, *H2-Ab1*, and *Irg1* were among the most downregulated genes (Figure 7b). On the other side, treatment with both compounds upregulated genes with potential neuroprotective function such as *Meg3*, *Sparcl1* and *Ppargc1a* (Figure 8b and Figure 9a). Interesting differences were also observed between the treatments: ORY-2001 but not FTY720 induced *Ttr* and the hemoglobin chain genes *Hba-a1* and *Hbb-b1* (Figure 8b and Figure 9a) and downregulated *Aif1* (Figure 9a), *Bst2*, *Rgs4*, and *Mtap9* in the spinal cord (Figure 9a). In addition, ORY-2001 but not FTY720 prevented the induction of *Ptpn11*, required for initial infiltration of pioneer CD^8+^ T-cells into the CNS in EAE [42], and of *Kif1b* and *Pik3r1*, associated with susceptibility to MS by GWAS [43] (Figure 9a,c). ORY-2001 also preserved the expression of *Ogn* (Figure 9a), a gene coding for an extracellular matrix protein reduced during demyelination [44] in EAE and downregulated by FTY720, and *Dcn*, *Mpz*, *Grik1*, and *Ecrg4* (Figure 9a). These differences were correlated with the greater damage induced by MOG_35–55_ in the animals treated with FTY720 than with ORY-2001, as reflected in the clinical score. Finally, we also reviewed *S100a9*, a biomarker identified previously to be modulated by ORY-2001 [12] and induced in both MS and EAE. *S100a9* was downregulated in the spinal cord by ORY-2001 in the effector phase (Figure 7b and Figure 9a). 

Gene expression changes in the brain were less prominent than in the spinal cord, but confirmed the anti-inflammatory effect of ORY-2001, a finding that was especially clear in the effector phase (Figure 7c,d and Figure 8c,d). ORY-2001 was more effective at reducing inflammatory markers than ORY-LSD1 in the sub-chronic phase (Figure 7f,g). In the effector phase, gene expression changes induced in the brain in EAE mice were also similar for ORY-2001 and FTY720 (r = 0.70) but more potent for the first (slope = 0.7308; Figure 9b). Downregulated genes included the acute phase proteins *Saa1* and *Saa3*; genes involved in antigen presentation (*H2-Eb1*, *H2-Ab1*, *H2-Aa*), complement (*C3*), chemokines (*Ccl2*, *Ccl5*, *Ccl8*), cytokine signaling (*Gbp2*, *Gbp3*, *Gbp6*), *Nmes1*, *TC1658796 (Mpeg1)*, *Irgm*, *Serpina3n*, *Oasl2*, *Timp1*, *Tgfbi*, *Lgals3* and *Cd52*. A remarkable difference in the sub-chronic relative to the effector phase was the downregulation in the brain of the secretoglobin *Scgb1c1*, possibly associated with the remission of symptoms. Strikingly, in the effector phase, both compounds potently downregulated the pituitary markers *Gh*, *Prl*, *Pomc*, and *Cga* (Figure 9b). The same genes were upregulated in the sub-chronic phase, while contrastingly inflammation associated markers remained downregulated. The only clear difference between FTY720 and ORY-2001 in the effector phase was the induction of *Cox1* by FTY720 and not by ORY-2001 (Figure 9b), although *Cox1* was also upregulated by ORY-2001 and ORY-LSD1 in the sub-chronic phase (Figure S4). 

A subset of markers identified in the microarray surveys was selected for validation by qRT-PCR on individual samples from spinal cord and brain. The mean expression values in individual samples, represented as −ΔΔCp (Figure 9c,d) or −ΔΔCt values (Supplementary Figure S4h,i), largely confirmed the regulation of the biomarkers observed in the microarray survey. For example, ORY-2001 downregulated the inflammatory biomarkers *Saa3*, *Aif1* and *Gbp2* in the spinal cord and the brain in the effector phase (Figure 9c,d); and *Ccl5*, *Ip10* and *Tnf-alpha* in the sub-chronic period in both target tissues (Supplementary Figure S4h,i).

*Gh*, *Prl*, *and Pomc* were upregulated in the brain of treated mice in the subchronic phase but downregulated in the effector phase, although only *Prl* and *Pomc* reached significance (Figure 9d). These three genes exhibited highly heterogeneous expression in individual vehicle-treated samples but homogeneously low expression levels in treated samples in the effector phase. It is known that release of *Gh* and *Prl* in plasma occurs in non-synchronized short-lasting bursts [45], and such volatility of expression levels may have contributed to the high variability observed in the vehicle group, however the homogenously low levels in the treated samples suggest an effective downregulation. Finally, Ttr, found upregulated by ORY-2001 in the spinal cord (Figure 9a), was discarded as a biomarker since qRT-PCR showed the increased value of the pooled sample analyzed in the microarray survey resulted from the contribution of a single outlier sample. 

### 3.7. ORY-2001 and FTY720 Act through Different Mechanisms of Action

**Figure 10:**
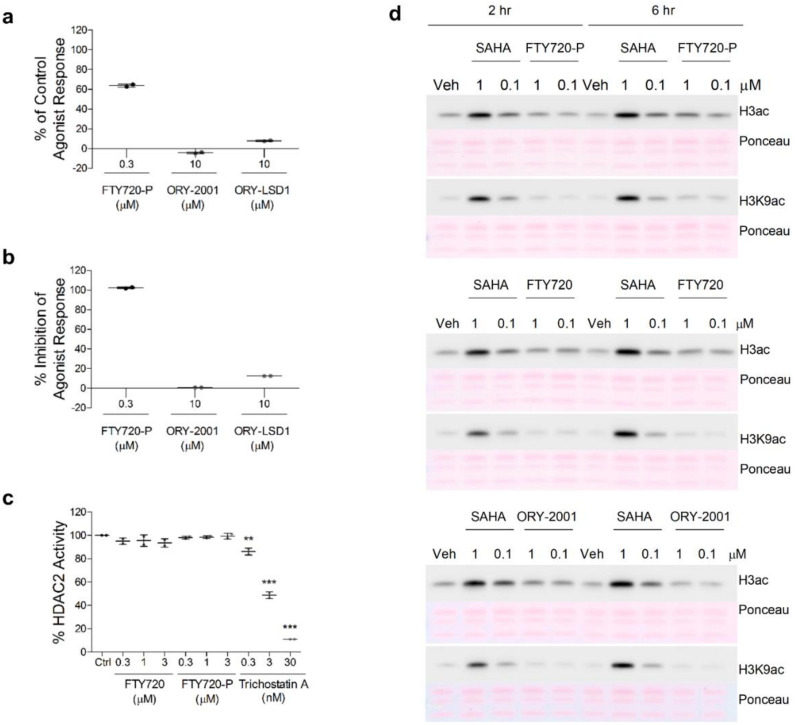
Effect of ORY-2001, ORY-LSD1, FTY720 and FTY720-P on S1PR and HDAC. Evaluation of the agonistic (**a**) and antagonist (**b**) effect of the compounds on the human S1P1 receptor. Data are expressed as the percentage of the impedance response of the control agonist (S1P at 0.3 μM; EC_50_ = 0.001 μM), and as the percentage of impedance inhibition of the control agonist response (S1P at the EC_80_ = 0.01 μM), *n* = 2; (**c**) HDAC2 biochemical activity. Data are expressed as percentage of control (no compound) activity, *n* = 2. TSA was used as a control; (**d**) Cellular HDAC activity assay. H3 acetylation (H3ac) levels detected by Western blot analysis using global and H3K9-specific antibodies on cell extracts of SH-SY5Y cells after 2 (left) and 6 h (right) treatment with FTY720-P (top), FTY720 (center) and ORY-2001 (bottom) at 1 and 0.1 µM. SAHA was used as a control. Ponceau S staining was used as loading control. Veh: Vehicle. Representative image of *n* = 3 (*n* = 4 for global H3ac and *n* = 1 for H3K9ac). ** *p* < 0.01, *** *p* < 0.001 between vehicle and treated sample. TSA: Trichostatin A. SAHA: suberoylanilide hydroxamic acid.

The observations that both ORY-2001 and FTY720 reduce lymphocyte egress from the periphery to the CNS, as well as the finding that they induce highly similar gene expression changes in brain and spinal cord, reveals the possibility that their mechanisms of action could be related. FTY720 becomes phosphorylated in vivo by the sphingosine kinases SPHK1 and SPHK2 and then acts as a sphingosine 1 phosphate receptor antagonist by binding to the S1P receptor and provoking its internalization and degradation [36]. While FTY720-P acts both as an agonist [46] and antagonist [47] in GPCR based impedance assays in cells expressing the S1P1 receptor, neither ORY-2001 nor ORY-LSD1 (at concentrations well above those needed to fully inhibit KDM1A) showed any activity in these assays (Figure 10a,b), hence the sphingosine 1 phosphate receptor is not a target of these KDM1A inhibitors. On the other hand, nuclear S1P and FTY720-P were reported to act as direct inhibitors of HDAC1/2 [48,49], proteins participating together with KDM1A in transcription regulation. If the FTY720 and ORY-2001 are indeed target components of the same transcriptional complex, this could explain why they produce such highly similar effects. However, we were unable to confirm the previous studies reporting that FTY720 or FTY720-P had any significant impact on recombinant HDAC2 or cellular HDAC activity as assessed by measuring acetylated H3 or H3K9 levels (Figure 10c,d). Hence, HDAC1/2 is not a target of FTY-720 and therefore ORY-2001 and FTY-720 appear to act through different mechanisms of action. 

## 4. Discussion

Despite decades of research, the etiology of MS is still unclear. Epidemiological studies suggest that part of the missing heritability and etiology of MS could be explained by epigenetics. Histone modifying enzymes are emerging as epigenetic targets of special interest for the development of novel therapies for patients with MS [50]. For example, HDAC inhibitors have been reported to cause a shift in dominance from the Th1 to Th2 lymphocyte phenotype, to expand anti-inflammatory Tregs, and to modulate the expression of cytokines like IL10 [51]. Chromatin remodeling mediated by the H3K27me2/3 demethylase KDM6B is important in the acquisition of the M2-macrophage phenotype [52]. The KDM6 inhibitor GSK-J4 has been described to inhibit Th17 cell differentiation in vitro [53] and to promote Tregs [54]. However, so far, no role has been described for the H3K4me1/2 demethylase KDM1A in MS.

Here, we characterized the immunomodulatory and neuroprotective effects of the new orally bioavailable and brain penetrant KDMA1 inhibitor ORY-2001 (vafidemstat). ORY-2001 was demonstrated previously to modulate genes required for synaptic plasticity. Vafidemstat rescued cognitive function and reduced the inflammatory signature in the hippocampus of SAMP8 mice, including T-cell receptor b genes and the expression of the alarmin S100a9 [20], an inflammatory biomarker upregulated in several CNS diseases with inflammatory component, including MS [55]. Compounds that disrupt binding of S100A9 to the Toll-Like Receptor 4 (TLR4) were shown to inhibit acute EAE in mice [56], we decided to evaluate the potential effect of vafidemstat in this autoimmune neurodegenerative model. We found that vafidemstat reduced lymphocyte egress, inflammation and the clinical score of the EAE model, and exerted its therapeutic action in a dose window similar to that observed previously in SAMP8 mice [20]. In the Theiler virus model, vafidemstat decreased the TMEV clinical score, reduced lymphocyte infiltration of immune cells in the spinal cord and glial activation, and improved axon integrity by preventing demyelination. In EAE mice, treatment reduced the MOG_35–55_-induced proliferation of immune cells without affecting the anti-CD3 induced response, illustrating that vafidemstat does not provoke general immune-suppression. The doses of vafidemstat used in the EAE and TMEV preclinical models do not significantly impact the total number of circulating lymphocytes in GLP toxicology studies (Supplementary Materials Table S1).

Our results suggest that a direct effect on the Th1/Th17 cell-driven encephalitogenic response is unlikely to drive the protective effect of vafidemstat as the treatment did not modify the production of the peripheral immune factors IFNγ or IL-17. Although the in vivo oral treatment with vafidemstat did not decrease the potential autoreactive response of T cells, a direct reduction of their encephalitogenic activity was exerted when the T cells were incubated in vitro with the compound at a dose sufficient to guarantee full inhibition. On the other hand, treatment with vafidemstat induced the accumulation of immune cells (significantly for the effector CD4 T cell population) in peripheral lymphoid organs, thus avoiding their recruitment to the CNS, which could be correlated with the effect of this compound in the chemokine production. 

Treatment with vafidemstat induced IL-4, characteristic of Th2 cells, which could critically contribute to its protective response [57]. The significant increase in the IgG1 antibody isotype also suggested an enhancement of the Th2-type response [58]. In addition, TNF-alpha, which is usually overproduced in MS, was significantly decreased by vafidemstat. This cytokine has an important role in initiating and limiting the extent and duration of immune-mediated inflammatory processes, organ damage, cell infiltration to the spinal cord, and demyelination [59,60,61,62,63]. Recently, it has been suggested that the downregulation of TNF could be associated with EAE remission [64,65]. Together, these findings support the induction of an anti-inflammatory environment in the spinal cord of EAE animals treated with vafidemstat that was crucial to avoid and/or protect against subsequent demyelination. 

The efficacy of vafidemstat in the EAE model was found to be driven by KDM1A inhibition. Vafidemstat exhibits a more potent and long-lasting effect than ORY-LSD1 in chronic EAE. The efficacy correlated with a more significant anti-inflammatory action and with a more efficient reduction of lymphocyte egress to the CNS. Vafidemstat recapitulates the main therapeutic and molecular effects of the oral MS drug fingolimod (FTY720) but was more effective and/or faster acting in the effector phase of the EAE model. In the effector phase, only vafidemstat significantly reduced the recruitment to encephalitogenic/inflammatory cells to CNS (mainly to the lumbar region of the spinal cord), improved the clinical course of the disease, and reduced the levels of autoantibodies favoring a Th2 response. 

FTY720 is phosphorylated and binds to the S1P receptors in the cell membrane of lymphocytes, inducing receptor internalization and degradation, and immune-modulatory effects [66]. Nuclear FTY720-P produced by SPHK2 [67] has been reported to inhibit HDAC1 and HDAC2 [49], proteins that participate in transcription regulatory complexes with KDM1A, targeted by vafidemstat. Although we were not able to confirm inhibition of HDACs by FTY720-P, another MS drug, dimethylfumarate (DMF), was reported to downregulate the expression of HDAC1/2 and increase NRF2 signaling [68,69]. The broad spectrum HDAC inhibitor valproic acid ameliorated clinical signs in EAE [70] and more recently T cell-specific deletion of HDAC1 was proven to prevent EAE [71]. Together, these data point at a convergence of the mechanism of action on an epigenetic hub with a central role for the KDM1A/RCOR1/HDAC1/2 complex for developing novel MS therapy strategies.

Using microarray-based surveys and qRT-PCR, we analyzed the gene expression changes in vafidemstat, ORY-LSD1 and FTY720 versus vehicle treated EAE mice in the subchronic and effector phase. All compounds induced a broad and highly robust reduction of the inflammatory markers induced by MOG in EAE mice. In line with the clinical score, the global expression changes induced by vafidemstat in the sub-chronic phase were more prominent than those induced by ORY-LSD1. In the effector phase, expression changes induced by vafidemstat were highly similar but more prominent than those induced by FTY720, confirming the previous observation that ORY-2001 may be a more potent or faster acting compound. 

Interestingly, among the genes downregulated most potently by vafidemstat treatment in the spinal cord in the effector phase, we identified a set of inflammatory mediators (*Ccl6*, *Sirpb1*, *Irg1*, *Arg1*, *Chi3l3* and *Ms4a8a*) that are selectively induced during the early phase of symptomatic development in EAE mice [41]. These transcripts may originate in a population of Arg1^+^ CNS myeloid cells unable to activate myelin specific T-cells described to arise around the peak of EAE, likely through an adaptive shift in expression of iNOS^+^ cells in response to the excessive inflammatory response and damage generated in EAE mice [71]. Thus, the downregulation of these genes by vafidemstat treatment may reflect reduced immune cell infiltration and/or lack of the need for this adaptative shift. Upregulation of *Arg1* and *Chi3l3*, a close homologue of *Chi3l1* (YKL-40) was also detected in mouse models for AD [72]. YKL40 represents a pathophysiological biomarker reflecting immune/inflammatory mechanisms in neurodegenerative diseases and it was found to be also increased in and/or associated with progression of patients with FTD, amyotrophic lateral sclerosis, neuromyelitis optica, and MS [73,74,75]. Of note, YKL40 levels in patients treated with vafidemstat in the AD Phase II trial ETHERAL were significantly reduced relative to placebo [76]. *Meg3*, *Sparcl1* and *Ppargc1a* presented the highest upregulation by vafidemstat in the spinal cord in the effector phase. PPARGC1A is an important cofactor of NRF2. While reduced neuronal PPARGC1A expression in the MS cortex was described to partly underlie mitochondrial dysfunction in MS grey matter and to contribute to neurodegeneration in MS cortex, increased expression in astroglia was proposed to be protective [77]. SPARCL1 and MEG3 are involved in synaptic maintenance or plasticity. SPARCL1 is produced in astrocytes and promotes excitatory synapse formation in vitro and in the developing nervous system in vivo. In EAE mice, paralysis severity correlates inversely with the ratio of the SPARCL1 to *Sparc* transcript, a related gene which antagonizes the synaptogenic action of SPARCL1 [78]. In addition, two single nucleotide polymorphisms (SNPs), rs9998212 and rs7695558, associated with lower brain *Sparcl1* gene expression, have been shown to accelerate AD pathogenesis [79]. MEG3 is a maternally expressed gene found downregulated in HD brains and regulated by REST/NRSF, a ZNF transcription factor known to recruit KDM1A and HDAC1/2 to repress its target genes [80]. We also found that in the brain some pituitary genes (*Gh*, *Prl*, *Pomc*, and *Cga)* were differentially regulated by vafidemstat during the progression of the disease: downregulated at the effector phase and upregulated at the sub-chronic period. KDM1A has been shown to be directly involved in the control of these genes during pituitary development, where opposing KDM1A recruiting complexes function initially in their developmental activation and later in their repression [81]. The regulation of *Gh* is particularly interesting since GH has been suggested to influence the function of the immune system. In fact, Gh deficient Ghrh KO mice do not develop EAE [82], suggesting that an initial downregulation or prevention of upregulation may be relevant to limit the damage. Vafidemstat also has inherent neuroprotective power, as illustrated by its capacity to induce the expression of neuroprotective genes and to protect motoneurons in spinal cord explants from glutamate induced excitotoxicity.

Vafidemstat is a new oral drug with potential therapeutic application for treatment of chronic MS. So far, very few oral drugs have been approved by the FDA for the treatment of this disease (fingolimod/FTY720, siponimod, DMF, cladribine, and teriflunomide) and none of them work in the primary progressive form of the disease [83,84,85,86,87]. In addition, currently used MS drugs have several undesired side effects. Unlike FTY720, vafidemstat does not target the S1PR’s and is therefore not expected to induce bradycardia, effects on blood pressure, or leakage of the blood-brain barrier associated with S1PR1 modulation [88,89,90]. In addition, vafidemstat provided effective immunomodulation at doses that did not significantly impact the total number of circulating lymphocytes and did not induce any signs of gastrointestinal toxicity, a side effect that has caused many patients to abandon treatment with DMF [91]. Also, the rapid mode of action of vafidemstat indicates that the compound may be appropriate to treat patients with acute MS flares or other inflammatory conditions, and that it may offer an alternative to corticosteroids in patients that have developed an allergy or resistance to this treatment. Finally, and in view of recent report pinpointing at the infection with Epstein Barr virus as one of the leading potential causes for MS, it is worth mentioning that inhibitors of KDM1A have been shown to be active against other members of the family of Herpesviridae [92]. 

So far, vafidemstat has been administered to more than 300 subjects in multiple studies and has been shown to be safe. Vafidemstat has been evaluated in a Phase I trial in healthy volunteers (EUDRACT Nº 2015-003721-33), and significantly reduced aggression in borderline personality disorder (BPD), attention deficit/hyperactivity disorder (ADHD), autism spectrum disorder (ASD) and in moderate-severe AD in the Phase IIa trials REIMAGINE and REIMAGINE-AD; EUDRACT Nºs 2018-002140-88 and 2019-001436-54 [93,94] and in mild to moderate AD (ETHERAL; EUDRACT Nº 2017-004893-32 and ETHERAL-US; NCT03867253). The first safety and efficacy data for the use of vafidemstat in relapse-remitting and secondary progressive MS (SATEEN; EUDRACT Nº 2017-002838-23) indicate that long-term vafidemstat treatment was safe and well tolerated and modulated the Th1/Th2 cytokines including a dose dependent decrease of IFNγ/IL4 ratio [95]. Finally, based on its anti-inflammatory activity reported here and favorable safety profile, vafidemstat has also been evaluated in a randomized, open–label Phase II study to evaluate its efficacy and tolerability of vafidemstat in combination with standard of care treatment to prevent Acute Respiratory Distress Syndrome (ARDS) in adult severely ill patients with COVID-19 (ESCAPE; EUDRACT N° 2020-001618-39) [96]. 

## 5. Conclusions

Vafidemstat decreases the clinical scores in the EAE and TMEV models, reduces lymphocyte egress and the infiltration of immune cells in the spinal cord and microglial activation, and improves axon integrity by preventing demyelination. 

The therapeutic effects of vafidemstat can be achieved at doses that do not significantly affect hematology or lymphocyte counts, a common side effect in MS drugs, and without signs of gastro-intestinal toxicity. 

KDM1A, an epigenetic target that forms part of the KDM1A/RCOR1/HDAC transcription regulatory complexes, is the prime target of vafidemstat in vivo.

HDAC inhibitors have been shown to improve clinical score in EAE mice and several FDA-approved drugs for the treatment of MS were reported to target HDAC, including the S1P receptor modulator FTY720 and fumarates. 

This points to a convergence of the mode of action of these drugs and highlights the relevance of the epigenetic axis to the therapeutic effect in MS models. 

Contrary to what was reported in previous studies, FTY720 did not inhibit HDAC.

Vafidemstat was more effective and/or faster acting than FTY720 in the effector phase in EAE mice. 

The gene expression response to vafidemstat in the spinal cord and brain of EAE mice reflects a potent down-regulation of the inflammatory response and is highly similar but more prominent than the response induced by FTY720.

Vafidemstat is a novel epigenetic drug that has demonstrated proof of concept in the EAE and Theiler models in mice. The compound has a favorable safety profile and is in Phase II of clinical development in multiple indications.

## 6. Patents

TM: CM and DR are listed as inventors of a patent application WO/2017/212061A1 assigned to Oryzon Genomics S.A. related to the content of this manuscript.

## Data Availability

The microarray gene expression dataset in this manuscript has been submitted as NCBI GEO GSE118071. Data have been uploaded in Mendeley Data (V1, doi:10.17632/xfbnyxvw8k.1).

## Supplementary Materials

The following supporting information can be downloaded at: https://www.mdpi.com/article/10.3390/pharmaceutics14071420/s1, Figure S1: ORY-2001 alleviates clinical symptoms of severe EAE; Figure S2: ORY-2001 reduces inflammatory infiltration and demyelination in spinal cord of EAE mice; Figure S3: Treatment with ORY-2001 of EAE mice provides higher protection from the CNS damage than FTY720; Figure S4: Gene expression analysis in brain and spinal cord of mice treated with ORY-2001, ORY-LSD1 or FTY720; Table S1: List of reagents and resources; Table S2: Primers and PCR conditions for qRT-PCR; Table S3: Genome wide treatment comparison statistics; Table S4: Effect of ORY-2001 on lymphocyte count.

## Abbreviations

7-AAD	7-amino-actinomycin D
AD	Alzheimer’s Diseases
APC	Allophycocyanin
ADHD	Attention deficit/hyperactivity disorder
ASD	Autism spectrum disorder
BSA	bovine serum albumin
CNS	Central nervous system
ConA	Concanavalin A
DLNs	Draining lymph nodes
DMEM	Dulbecco’s Modified Eagle Medium
DMF	Dimethylfumarate
EAE	Experimental autoimmune encephalomyelitis
FBS	Fetal bovine serum
FCS	Fetal Calf Serum
FTY720	Fingolimod
GBSS	Gey’s balanced salt solution
HACTV	Horizontal activity
HBSS	Hank’s Balanced Salt Solution
HDAC1/2	Histone deacetylase 1 and 2
HPβCD	hydroxypropyl-β-cyclodextrin
IC_50_	Inhibitory concentration 50
KDM1A	Lysine (K)-specific demethylase 1A
LSD1	Lysine specific demethylase
MAO-B	Monoamine oxidase B
MEM	Minimal essential medium
MOG35–55	Myelin oligodendrocyte glycoprotein
MPP+	1-methyl-4-phenylpyridinium
MPTP	1-methyl-4-phenyl-1,2,3,6-tetrahydropyridine
MS	Multiple sclerosis
OCT	Optimal cutting temperature compound
ORY-1001	Iadademstat
ORY-2001	Vafidemstat
ORY-LSD1	LSD1 selective inhibitor
PBS	Phosphate bufferd saline
PE	Phycoerythrin
PFU	Plaque forming units
PVP-40	Polyvinylpyrrolidone-40
RCOR	REST corepressor
S1PRs	Sphingosine 1 phosphate receptors
SAMP8	Senescence accelerated mouse prone 8
SAHA	Suberoylanilide hydroxamic acid
TBS	Tris buffered saline
TCP	Tranylcypromine
THA	DL-threo-β-hydroxyaspartic
TLR4	Toll-Like Receptor 4
TMEV	Theiler’s murine encephalomyelitis virus
TSA	Trichostatin A
VACTV	Vertical activity
