# Supplementary material for: Efficacy of Vafidemstat in Experimental Autoimmune Encephalomyelitis Highlights the KDM1A/RCOR1/HDAC Epigenetic Axis in Multiple Sclerosis
Source: Pharmaceutics. 2022 Jul 6;14(7):1420. doi: 10.3390/pharmaceutics14071420 (PMC9323733; doi:10.3390/pharmaceutics14071420)
Supplement: Supplementary file 1 [file pharmaceutics-14-01420-s001.zip › pharmaceutics-1778942-supplementary.pdf]

Supplementary Materials: Efficacy of vafidemstat in experimental autoimmune encephalomyelitis highlights the KDM1A/RCOR1/HDAC epigenetic axis in multiple sclerosis

Fernando Cavalcanti[1,#], Elena Gonzalez-Rey[2,#], Mario Delgado[2], Clara P. Falo[2], Leyre Mestre[3], Carmen Guaza[3], Francisco O´Valle[4], Michele MP Lufino[1], Jordi Xaus[1], Cristina Mascaró[1], Serena Lunardi[1], Natalia Sacilotto[1], Paola Dessanti[1], David Rotllant[1], Xavier Navarro[5], Mireia Herrando-Grabulosa[5], Carlos Buesa[1] and Tamara Maes[1,*]

[1] Oryzon Genomics, S.A. Carrer Sant Ferran 74, 08940 Cornellà de Llobregat, Spain
[2] Institute of Parasitology and Biomedicine Lopez-Neyra, IPBLN-CSIC, PTS-Granada, Granada, Spain
[3] Department of Functional and Systems Neurobiology, Cajal Institute (CSIC), Madrid, Spain.
[4] Department of Pathology, School of Medicine, IBIMER and IBS-Granada, Granada University, Spain
[5] Departament de Biologia Cellular, Fisiologia i Immunologia, Institut de Neurociències, Universitat Autònoma de Barcelona, and Centro de Investigación Biomédica en Red sobre Enfermedades Neurodegenerativas (CIBERNED), Bellaterra, Spain.

* Correspondence: tmaes@oryzon.com
\# Both authors equally contributed to this study.

Sex and Gender in this Research

Multiple sclerosis (MS) is more prevalent in women than men, but male patients develop more severe clinical symptoms and deteriorate faster than female patients. With respect to the animal models for MS used in this research, the following aspects were considered:

Drug Exposure:
Minor differences were observed between the exposure of ORY-2001 in male and female mice and rats, which were not deemed relevant for this research. Previous efficacy studies in the SAMP-8 model did not indicate a difference in the therapeutic doses for ORY-2001 in females vs male cohorts [20]. In view of these data, it was not justified to increase the number of animals usedand perform separate studies for male an female mice in MS models.

MOG_{35-55} induced EAE in C57BL/6 mice:
No differences have been observed in disease incidence or severity between sexes in MOG_{35-55} induced EAE in C57BL/6 mice in a study by Papenfuss et al. (J Neuroimmunol, 2004;150(1-2):59-69, therefore it was not deemed justified to increase the number of animals used in the studies to study males and females independently in the experiments reported in this manuscript.

TMEV in SJL mice:
Although a higher incidence and severity has been reported for male than female SJL mice in the TMEV model (Alley et al. Exp Neurol, 2003 Mar;180(1):14-24), females are used in most studies because the strain of SJL male mice are very aggressive and display a high level of dominance and inflict wounds to their housemates. Male cohorts are not not suitable for chronic models (PLoS One. 2021 May 14;16(5):e0251416).

Spinal cord organotypic cultures from 8 day old Sprague-Dawley pups:
Hypothetical sexual dimorphism in spinal cord organotypic cultures and chronic excitotoxicity treatment derived from 8 day old Sprague-Dawley male or female pups was not assessed. Sexual dimorphism in the neurons of the rat spinal cord has been known for a long time. The motoneurons in the spinal nucleus of the bulbocavernosus muscle, involved in copulatory movements, have been known to develop under neonatal exposure to testosterone: as a consequence male rats have many more SNB motoneurons than do females (Breedlove, S. M. and Arnold, A. P. Science , 1980; 210:564-566). However, samples for organotypic cultures were obtained at very early stage, did not include the SNB, and sexual dimorphism was therefore unlikely to affect this study, and it was not justified to increase the number of animals used in the study to study males and females independently in the research reported in this manuscript.

Table S1: List of reagents and resources

REAGENT OR RESOURCE	SOURCE	IDENTIFIER
**Antibodies**		
Anti-IgG1 (Biotin-labeled)	Serotec	Cat# 107008
Anti-IgG2a (Biotin-labeled)	Serotec	Cat# 108008
Anti-IL-4 (Purified Rat Anti-Mouse)	BD Pharmingen	Cat# 554387
Anti-IL-4 (Biotin Rat Anti-Mouse)	BD Pharmingen	Cat# 554390
Anti-TNF-alfa (Purified Rat Anti-Mouse)	BD Pharmingen	Cat# 559064
Anti-TNF-alfa (Biotin Rat Anti-Mouse)	BD Pharmingen	Cat# 558415
Anti-IFN-gamma (Purified Rat Anti-Mouse)	BD Pharmingen	Cat# 554030
Anti-IFN-gamma (Biotin Rat Anti-Mouse)	BD Pharmingen	Cat# 551506
Anti-IP-10 (Anti-Murine Purified PolyclonalAntibody)	Peprotech	Cat# 500-P129
Anti-IP-10-alfa (Biotin Rat Anti-Mouse)	Peprotech	Cat# 500-P129Bt
Anti-MCP-1 (Anti-Murine Purified PolyclonalAntibody)	Peprotech	Cat# 500-P113
Anti-MCP-1 (Biotin Rat Anti-Mouse)	Peprotech	Cat# 500-P113Bt
Anti-CD4 (PerCP/Cy5.5 conjugated)	BD Pharmingen	Cat# 550954
Anti-CD62L (APC conjugated)	BD Pharmingen	Cat# 559792
Anti-CD44 (PE conjugated)	BD Pharmingen	Cat# 550989
Anti-SMI-32 (Anti-Murine Purified MonoclonalAntibody)	Biolegend	Cat#801701
Donkey anti-mouse IgG (H+L)	Invitrogen	Cat#A21202
Anti-IBA-1 (AIF-1) antibody	Wako	Cat#019-19741
Anti-H3K9 Ac	Abcam	Cat# ab4441
Anti-H3 K9+K14+K18+K23+K27 Ac	Abcam	Cat# ab47915
HRP-donkey anti Rabbit IgG	Life Technologies	Cat# 31458
**Biological samples**		
EAE samples: Blood, spleen, lymph nodes, brainand spinal cord	Institute of Parasitology and Biomedicine Lopez-Neyra, IPBLN-CSIC,	N/A

	Granada, Spain.	
Theiler virus model samples: spinal cord	Department of Functional and Systems Neurobiology, Cajal Institute (CSIC), Madrid, Spain.	N/A
**Chemical, peptides and recombinant proteins**		
ORY-2001	Oryzon Genomics S.A.	Example 35 in WO2012/013728, purity >98%
ORY-LSD1	Oryzon Genomics S.A.	1R,2S of example 20 in WO2013/057320, purity > 98%
FTY720	Cayman Chemical	Cat# 10006292
FTY720	Selleckchem	Cat# S5002
rasagiline	Waterstone	Cat# WS104114
FTY720-P	Cayman Chemical	Cat# 10008639
Sphingosine 1-phosphate (S1P)	Sigma-Aldrich	Cat# S9666
VPC23019	Avanti Polar Lipids	Cat# 857360P
Trichostatin A (TSA)	Selleckchem	Cat# S1045
HDAC2 (Recombinant Human)	BPS Bioscience	Cat# 50002
IL-4 (Recombinant Mouse)	BD Pharmingen	Cat# 550067
TNF-alpha (Recombinant Mouse)	BD Pharmingen	Cat# 554589
IFN-gamma (Recombinant Mouse)	BD Pharmingen	Cat# 554587
IP10 (Recombinant Mouse)	Peprotech	Cat# 250-16
MCP-1 (Recombinant Mouse)	Peprotech	Cat# 250-10
DL-threo-β-hydroxyaspartic acid	Sigma-Aldrich	Cat#H2775
Riluzole	Sigma-Aldrich	Cat# R116-25MG
SAHA	Selleckchem	Cat# S1047
**Critical commercial assays and reagents**		
RNAeasy Mini Kit	Qiagen	Cat# 74106
High Capacity RNA to cDNA Master Mix	Thermo Fisher Scientific	Cat# 4390779
MessageAmp II aRNA Amplification Kit	Applied Biosystem	Cat# AM1751
EpiQuik Total Histone Extraction Kit	Epigentek	Cat# OP-0006-100

DMEM/F12 (HAM) 1:1	Sigma-Aldrich	Cat# D5671 and N4888
L-glutamine	LabClinics	Cat# GLN-B
Fetal Bovine Serum	Sigma-Aldrich	Cat# F9665-500ml
Fetal Bovine Serum	Invitrogen	Cat# 26400-044
NuPAGE MOPS SDS Running Buffer (20x)	Life Technologies	Cat# NP0001-102
NUPAGE 12% BT GEL 1.0MM 12W	Life Technologies	Cat# NP0342BOX
iBlot 2 NC Regular Stacks	Life Technologies	Cat# IB23001
Ponceau S solution	Sigma-Aldrich	Cat# P7170-1L
ECL Prime Western Blotting Detection Reagent	Sigma-Aldrich	Cat# GERPN2232
Dimethyl sulfoxide (DMSO)	Sigma-Aldrich	Cat# 154938
HDAC Assay Buffer	BPS Bioscience	Cat# 50031
Fluorogenic HDAC substrate 3	BPS Bioscience	Cat# 50037
HDAC Assay Developer	BPS Bioscience	Cat# 50030
Bovine Serum Albumin (BSA)	Fisher Scientific	Cat# BP9706100
Bovine Serum Albumin (BSA)	Sigma-Aldrich	Cat# A0281
HBSS buffer	Invitrogen	Cat# 14025
HEPES	Invitrogen	Cat# 15630
**Deposited data**		
Microarray gene expression dataset	This manuscript	GEO GSE118071
Raw data	This manuscript	Deposited at Mendeley with doi:10.17632/xfbnyxvw8k.1.
**Experimental models: cell lines**		
Human: SH-SY5Y	ATCC	Cat# CRL-2266
**Experimental models: organisms**		
EAE model: female C57BL/6 mice	Harlan Interfauna Iberica	N/A
Theiler's model: female JL/J mice	Charles River	N/A
Spinal cord organotypic THA: Sprague-Dawley rat	Servei Estabulari UAB	OFA
**Oligonucleotides**		
Aif1/Iba1	ThermoFisher Scientific	Mm00479862_g1
Amph	ThermoFisher Scientific	Mm01164382_m1
Ccl19	ThermoFisher Scientific	Mm00839967_g1
Ccl6	ThermoFisher Scientific	Mm01302419_m1
Cox1	ThermoFisher Scientific	Mm04225243_g1

Gapdh	ThermoFisher Scientific	Mm99999915_g1
Gbp2	ThermoFisher Scientific	Mm00494576_g1
Gh	ThermoFisher Scientific	Mm00433590_g1
Gusb	ThermoFisher Scientific	Mm03003537_s1
Meg3	ThermoFisher Scientific	Mm00522599_m1
Ogn	ThermoFisher Scientific	Mm00627199_m1
Pik3r1	ThermoFisher Scientific	Mm01282781_m1
Pls3	ThermoFisher Scientific	Mm00521302_m1
Pomc	ThermoFisher Scientific	Mm00435874_m1
Ppargc1a	ThermoFisher Scientific	Mm01208835_m1
Prl	ThermoFisher Scientific	Mm00599950_m1
Ptpn11	ThermoFisher Scientific	Mm00448434_m1
S100a9	ThermoFisher Scientific	Mm00656925_m1
Saa3	ThermoFisher Scientific	Mm00441203_m1
Sparcl1	ThermoFisher Scientific	Mm00447784_m1
Ttr	ThermoFisher Scientific	Mm00443267_m1
Uchl1	ThermoFisher Scientific	Mm00495900_m1
See Table S2 for more information on custom-made DNA primers used in this study	N/A	N/A
**Software and algorithms**		
Feature Extraction software (v10.1)	Agilent	https://www.agilent.com
Polyphemus	Oryzon Genomics S.A.	N/A
Prism 5.0	GraphPad	https://www.graphpad.com/
Keisan Online Calculator service	Casio	https://keisan.casio.com/calculator
ImageJ	NIH	https://imagej.nih.gov/ij/

Table S2. Primers and PCR conditions for qRT-PCR

Gene	GenBank Accesion #	Primer sequence	PCR conditions
Tnf-alpha	NM_013693.3	Sense: GCG ACG TGG AAC TGG CAG AAG AG<br>Antisense: TGA GAG GGA GGC CAT TTG GGA AC	64ºC-30 sec-35x
Il-1β	NM_008361.4	Sense: CTC CAT GAG CTT TGT ACA AGG<br>Antisense: TGC TGA TGT ACC AGT TGG GG	60ºC-45 sec-35x
Ip10	NM_ 021274.2	Sense: TGC CCA CGT GTT GAG ATC AT<br>Antisense: GGG TAA AGG GGA GTG ATG GA	60ºC-30 sec-35x
Il-4	NM_021283.2	Sense: AGC TAG TTG TCA TCC TGC TC<br>Antisense: AGT GAT GTG GAC TTG GAC TC	57ºC-1 min-35x
Rantes/Ccl5	NM_013653.3	Sense: CAT ATG GCT CGG ACA CCA CT<br>Antisense: GCG GTT CCT TCG AGT GAC AA	58ºC-30 sec-35x
Gapdh	XM_017321385.1	Sense: AAC TTT GGC ATT GTG GAA GG<br>Antisense: ACA CAT TGG GGG TAG GAA CA	Same as gene tested

Table S3: Genome wide treatment comparison statistics

Effector	Comparison	r^2	r	df	t	p value
**Spinal cord**	ORY-2001/FTY720	0.67	0.82	19489	199.9794	6.10E-4725
	ORY-2001/Veh	0.19	0.43	19489	66.5940	2.98E-870
	FTY720/Veh	0.23	0.48	19489	76.5470	4.23E-1115
**Brain**	ORY-2001/FTY720	0.49	0.70	19489	137.8720	9.92E-2884
	ORY-2001/Veh	0.14	0.38	19489	56.7222	3.52E-649
	FTY720/Veh	0.25	0.50	19489	80.8773	2.00E-1227

Microarray surveys were performed on pooled samples: Spinal Cord - N=5 mice/group (Vehicle, ORY-2001), N=4 (FTY720); Brain - N=10 mice/group (Vehicle, ORY-2001), N=6 (FTY720). Each datapoint was analyzed in triplicate. Correlation between mean gene expression changes induced by different treatments was analyzed calculating the Pearson correlation coefficient r using the Log2(Treatment/Veh) values for all genes expressed above background level (n = 19491). The t values for the student's t test werecalculated as: r x (df)1/2 x (1 – r2)-1/2; with the degrees of freedom df = n - 2. The two-tailed p values were calculated as 2 x tcd(t,df) using the t distribution function with the Keisan Online Calculator service (https://keisan.casio.com/calculator).

Table S4. Effect of ORY-2001 on lymphocyte count

**Treatment**<br>(mg/kg p.o. gavage)	**N**	**Lymphocytes**<br>(10^9/L)	**Equivalent mouse dose**<br>(mg/kg p.o gavage)
**28 d Wistar rats**			
Vehicle	10	3.56 ± 0.79	
ORY-2001 (0.07)	10	3.65 ± 1.39 (p=0.9947)	0.14
ORY-2001 (0.20)	10	3.44 ± 0.62 (p=0.8977)	0.40
ORY-2001 (0.60)	10	3.89 ± 1.14 (p=0.8152)	1.20
**6 months Wistar rats**			
Vehicle	10	3.57 ± 0.85	
ORY-2001 (0.07)	10	3.25 ± 0.75 (p=0.7872)	0.14
ORY-2001 (0.20)	10	4.02 ± 0.99 (p=0.5793)	0.40
ORY-2001 (0.40)	10	3.72 ± 1.12 (p=0.9698)	0.80
**28 d Beagle dogs**			
Vehicle	5	2.89 ± 0.48	
ORY-2001 (0.025)	3	3.31 ± 0.41 (p=0.8354)	0.15
ORY-2001 (0.075)	3	2.88 ± 1.02 (p=0.9999)	0.45
ORY-2001 (0.225)	5	4.33 ± 1.08 (p=0.0431)	1.35
**9 months Beagle dogs**			
Vehicle	6	2.20 ± 0.40	
ORY-2001 (0.025)	4	2.10 ± 0.60 (p=0.9925)	0.15
ORY-2001 (0.075)	4	2.90 ± 0.30 (p=0.3090)	0.45
ORY-2001 (0.150)	6	2.75 ± 1.04 (p=0.4075)	0.90

Effect of treatment with ORY-2001 administered by oral gavage on lymphocytes observed in GLP toxicological studies in rat and dog. Animals were treated orally (five consecutive days/week) by gavage 28 days or 6 months (rats) and 28 days or 9 months (dogs). Data represent de mean ± SD. The equivalent dose in mouse is indicated. 1-way ANOVA and Dunnett's Multiple Comparison test was used.

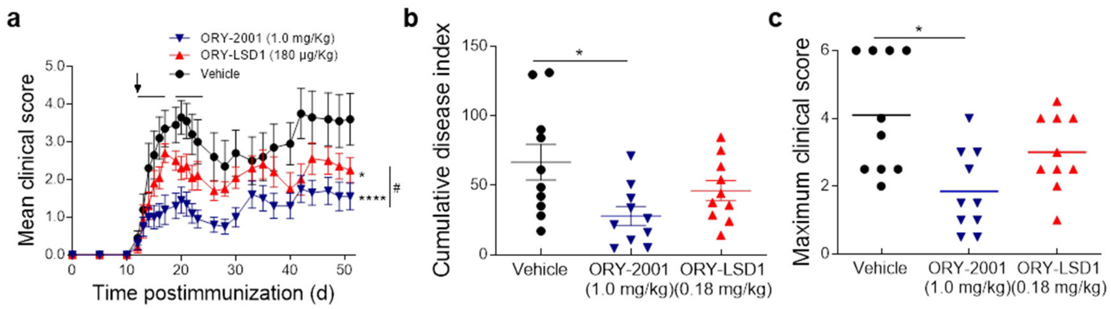

Figure S1. ORY-2001 alleviates clinical symptoms of severe EAE. (**a**) Chronic progressive EAE was induced in C57BL/6 mice by immunization with MOG_{35-55}. Immunized animals were treated orally during two periods of five days (horizontal bars) with vehicle, with ORY-2001 (1.0 mg/kg), or with ORY-LSD1 (180 μg/kg) starting at the onset on day 12 and sacrificed 52 days after immunization. The data represent the progression of the disease evaluated as the mean clinical score. *$p < 0.05$, ****$p < 0.0001$ between vehicle and treated mice; #$p < 0.05$ between ORY-2001 and ORY-LSD1 treatments. (**b**) The cumulative disease index (the sum of the clinical scores reached for each animal every day until day 51 post-immunization) and (**c**) the maximum clinical score reached by each animal in any day during the entire period investigated are showed in each experimental group (horizontal line represents the mean). n = 10 mice/group. *$p < 0.05$ between vehicle and ORY-2001-treated EAE mice. All data represent mean ± SEM.

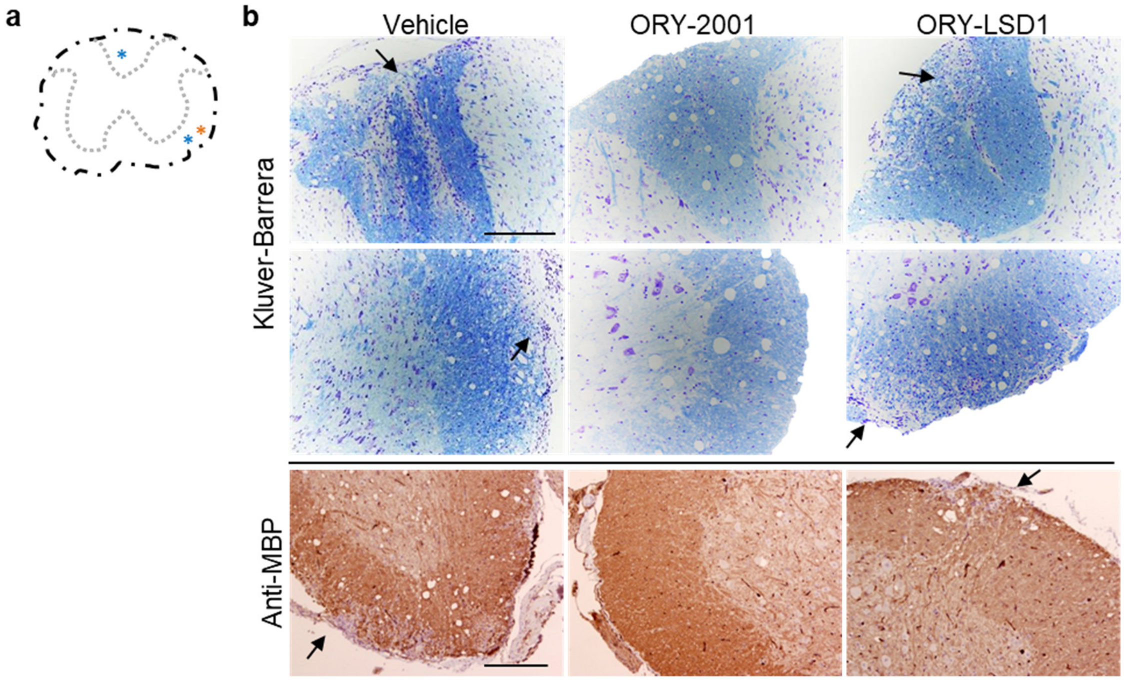

Figure S2. **ORY-2001 reduces inflammatory infiltration and demyelination in spinal cord of EAE mice.** Mice with $MOG_{35\text{-}55}$-induced chronic EAE were orally treated for two weeks with vehicle, ORY-2001 (0.5 mg/kg) or ORY-LSD1 (180 µg/kg) starting at the onset on day 12. Spinal cords were isolated immediately after treatment at the sub-chronic phase (26 days post-immunization) and processed for histopathological analysis of the disease. (**a**) Schematic representation of transverse sections of cervical spinal cord indicating specific regions cell infiltration (blue stars) or reduced myelin (brown star) in **b**. (**b**) Spinal cord sections were randomly selected in each group and stained with Klüver-Barrera or immunostained for myelin content with anti-MBP. Arrows point to areas of demyelination and inflammatory infiltration (as indicated in **a**). Images are representative of 5 mice/group. Scale bars: 200 µm.

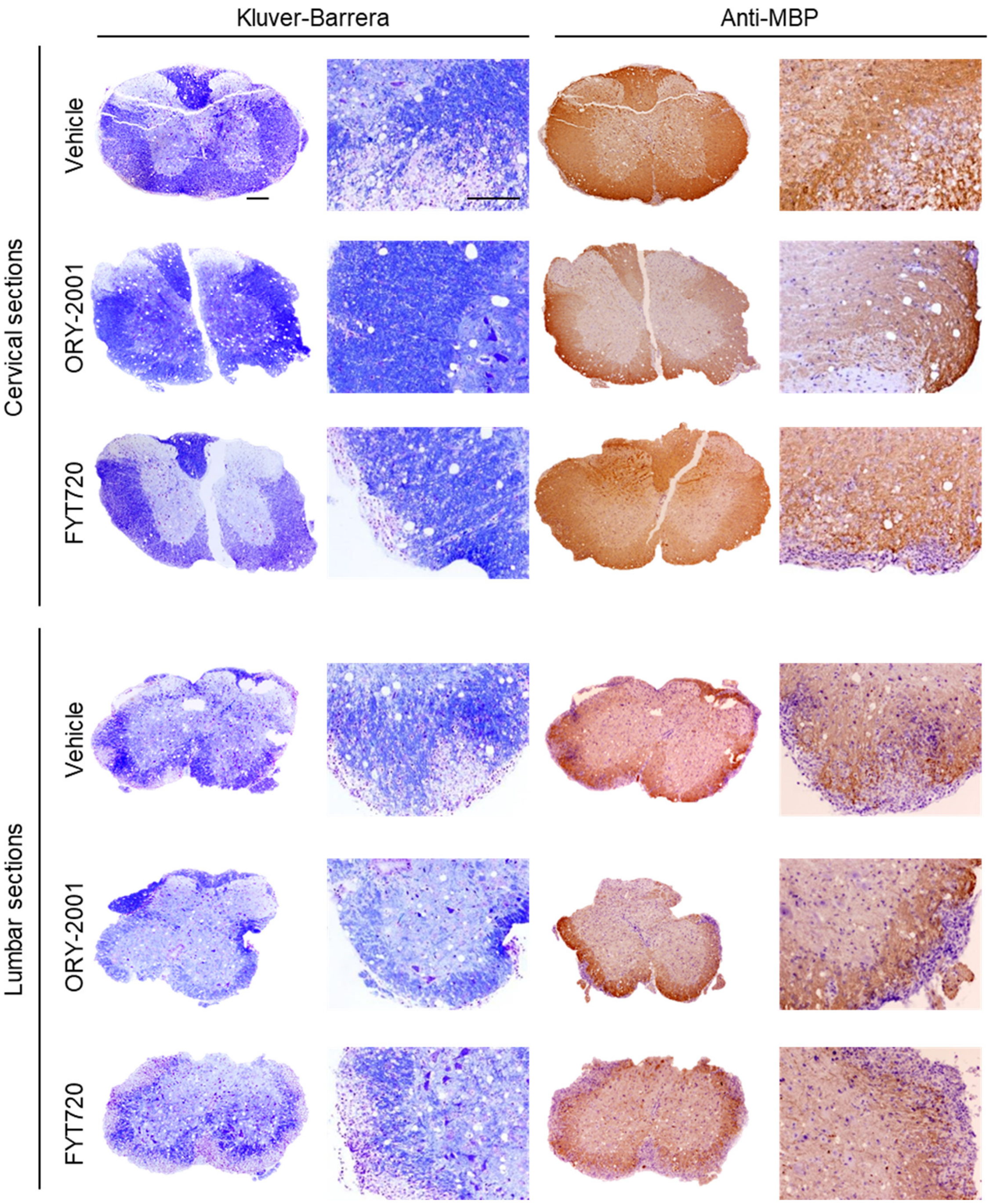

Figure S3. Treatment with ORY-2001 of EAE mice provides higher protection from the CNS damage than FTY720. Mice with MOG_{35-55}-induced chronic EAE were orally treated with vehicle, ORY-2001 (0.5 mg/kg) or FTY720 (1.0 mg/kg) for five consecutive days starting at the onset of EAE on day 12 and animals were sacrificed 24h after last treatment (corresponding to the peak of the disease, effector phase). Transverse sections of cervical (upper images) and lumbar (lower images) spinal cord randomly selected at the peak of chronic mild-EAE (17 days post-immunization) were stained with Klüver-Barrera (left images) and for myelin content (anti-MBP, right images) to detect areas of demyelination and inflammatory infiltration. Images are representative of 5 mice/group. Scale bars: 200 µm.

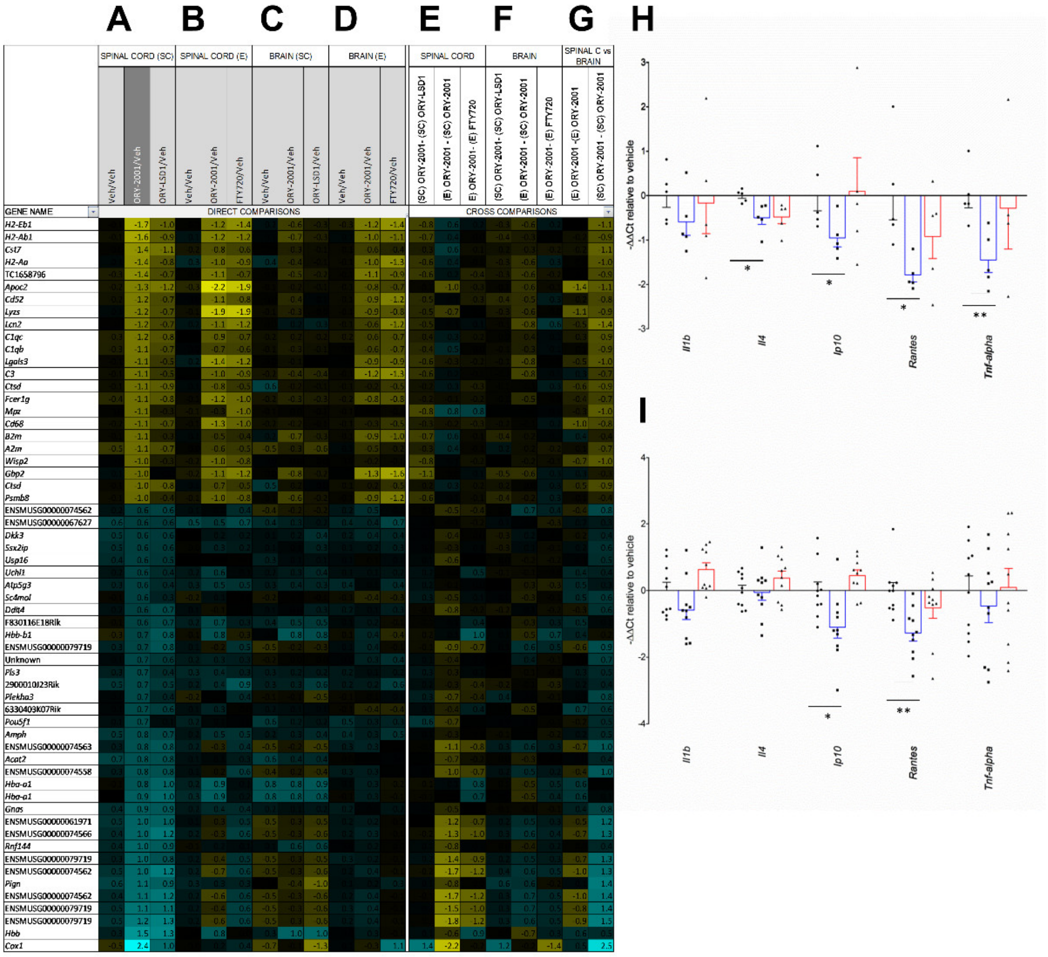

Figure S4. Gene expression analysis in brain and spinal cord of mice treated with ORY-2001, ORY-LSD1 or FTY720.

Animals were treated with Vehicle or ORY-2001 at 0.5 mg/kg, ORY-LSD1 at 0.18 mg/kg and FTY720 at 1 mg/kg (**a**) Microarray survey of gene expression changes in the spinal cord in the sub-chronic phase; 3 days after the last dose. (**b**) Gene expression changes in spinal cord in the effector phase. (**c**) Gene expression changes in brain in the sub-chronic phase; 3 days after the last dose. (**d**) Gene expression changes in spinal cord in the effector phase. (**e, f**) Comparison of the effect of the different treatments and disease phases in spinal cord (**e**) and brain (**f**). (**g**) Comparison of the effects of ORY-2001 in spinal cord and brain, in both phases. Pooled RNA from n = 5 spinal cords or 10 brains of Vehicle, ORY-2001 or ORY-LSD1 treated mice or from n = 4 spinal cords or 6 brains of FTY720 treated mice was used to perform each survey, n = 3 replicate probes within an array. Gene expression changes are expressed as Log_2(Treatment/Veh) for direct comparisons, and as the difference of the Log_2(Treatment/Veh) values for the respective conditions in the cross-comparisons. Genes up-regulated > 1.5 fold (Log_2(ORY-2001/Veh) > 0.6) or down-regulated > 2 fold (Log_2(ORY-2001/Veh) < -1) by ORY-2001 in the spinal cord in the sub-chronic phase were selected and represented for all comparisons. (**h, i**) qRT-PCR validations of selected individual genes modulated by ORY-2001 (blue), and ORY-LSD1 (red) in the sub-chronic phase in **h**) spinal cord and **i**) brain. Data are represented as $-\Delta\Delta$Ct values and mean ± SEM. (**h**) N = 5 mice/group, three technical replicate PCRs per mouse sample. (**i**) N = 10 mice/group, three technical replicate PCRs per mouse sample. Statistical analysis between Vehicle and ORY-2001 was calculated using two-tailed unpaired t-test. Welch's t-test correction

was applied when the populations had unequal variances. *$p < 0.05$, **$p < 0.01$ between vehicle and treated mice.
